# Mitochondrial metabolites extend lifespan

**DOI:** 10.1111/acel.12439

**Published:** 2016-01-05

**Authors:** Robert J. Mishur, Maruf Khan, Erin Munkácsy, Lokendra Sharma, Alex Bokov, Haley Beam, Oxana Radetskaya, Megan Borror, Rebecca Lane, Yidong Bai, Shane L. Rea

**Affiliations:** ^1^ The Barshop Institute for Longevity and Aging StudiesUniversity of Texas Health Science Center at San AntonioSan AntonioTX78229USA; ^2^ Department of PhysiologyUniversity of Texas Health Science Center at San AntonioSan AntonioTX78229USA; ^3^ Department of Cellular & Structural BiologyUniversity of Texas Health Science Center at San AntonioSan AntonioTX78229USA; ^4^ Department of Epidemiology and BiostatisticsUniversity of Texas Health Science Center at San AntonioSan AntonioTX78229USA; ^5^ Biotechnology ProgrammeCenter for Biological SciencesCentral University of South BiharPatna800014India

**Keywords:** α‐ketoglutarate‐dependent hydroxylases, aging, *Caenorhabditis elegans*, EGL‐9/PHD, glutaric acidemia, hypoxia‐inducible factor‐1, hypoxia‐inducible factor *isp‐1*, jumonji domain‐containing, metabolism, Mit mutants, mitochondria

## Abstract

Disruption of mitochondrial respiration in the nematode *Caenorhabditis elegans* can extend lifespan. We previously showed that long‐lived respiratory mutants generate elevated amounts of α‐ketoacids. These compounds are structurally related to α‐ketoglutarate, suggesting they may be biologically relevant. Here, we show that provision of several such metabolites to wild‐type worms is sufficient to extend their life. At least one mode of action is through stabilization of hypoxia‐inducible factor‐1 (HIF‐1). We also find that an α‐ketoglutarate mimetic, 2,4‐pyridinedicarboxylic acid (2,4‐PDA), is alone sufficient to increase the lifespan of wild‐type worms and this effect is blocked by removal of HIF‐1. HIF‐1 is constitutively active in *isp‐1(qm150)* Mit mutants, and accordingly, 2,4‐PDA does not further increase their lifespan. Incubation of mouse 3T3‐L1 fibroblasts with life‐prolonging α‐ketoacids also results in HIF‐1α stabilization. We propose that metabolites that build up following mitochondrial respiratory dysfunction form a novel mode of cell signaling that acts to regulate lifespan.

## Introduction

Several interventions have been described that extend the lifespan of *C. elegans*; these include dietary restriction, inhibition of protein translation, germ line ablation, and blockade of the insulin‐like signaling pathway (Lapierre & Hansen, 2012). An additional intervention is reduction of mitochondrial electron transport chain (ETC) activity (Munkacsy & Rea, 2014). In such mitochondrial (Mit) mutants, lifespan can be extended up to threefold (Rea, 2005). Both genetic and RNAi‐mediated disruption of mitochondrial respiratory components extends lifespan in *C. elegans*, but the mechanisms involved do not fully overlap—even when targeting the same gene (Yang & Hekimi, 2010b; Nargund *et al*., 2012; Munkacsy & Rea, 2014; Yee *et al*., 2014). These findings illustrate that more than one lifespan response pathway is activated in Mit mutants.

Previous studies have revealed that RNAi‐mediated knockdown of mitochondrial ETC components in a single tissue can increase the lifespan of otherwise wild‐type animals (Durieux *et al*., 2011). These studies also showed that ETC disruption in neuronal tissue was sufficient to induce a mitochondrial unfolded protein response (UPR^mt^) in untargeted intestinal cells. More recently, an octopamine‐dependent signal, originating in two head neurons, and/or the gonad, was shown to be sufficient to alter mitochondrial morphology in distal muscle, and this effect could be phenocopied by the addition of octopamine to wild‐type worms (Burkewitz *et al*., 2015). Whether octopamine functioned in a neural or endocrine role (Noble *et al*., 2013) remains unclear, but together these findings raise the intriguing possibility that lifespan regulation in Mit mutants may be mediated by one or more diffusible factors.

**Figure 1:**
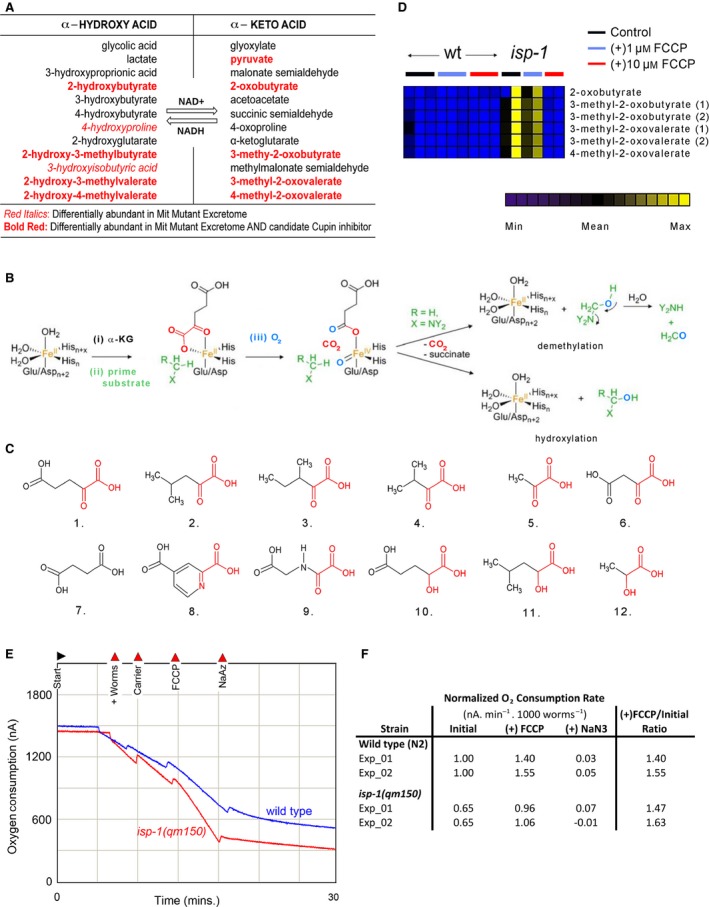
Mit mutants accumulate multiple α‐ketoacids that can be blocked by FCCP administration. (A) Various α‐ketoacids, and their reduced α‐hydroxyacids, accumulate in the exometabolism of Mit mutants [data extracted from (Butler *et al*., 2012)]. (B) Proposed catalytic mechanism of α‐ketoglutarate‐dependent hydroxylases. Figure is modified from (McDonough *et al*., 2010). (C) Structure of α‐ketoacids and other compounds relevant to the current study. (1) α‐ketoglutarate, (2) 4‐methyl‐2‐oxovalerate, (3) 3‐methyl‐2‐oxovalerate, (4) 3‐methyl‐2‐oxobutyrate, (5) pyruvate, (6) oxaloacetate, (7) succinate, (8) 2,4‐pyridinedicarboxylic acid, (9) N‐oxalylglycine, (10) 2‐hydroxyglutarate, (11) 2‐hydroxy‐4‐methylvalerate, (12) lactate. α‐ketoacid‐ and α‐hydroxyacid‐moieties are shown in *red*. (D) Blockade of α‐ketoacid accumulation in the exometabolome of *isp‐1(qm150)* Mit mutants following FCCP administration. Data is presented as a heat map showing the relative abundance of each metabolite across independent samples (columns). (E, F) Effect of FCCP on oxygen consumption by *isp‐1(qm150)* and wild‐type (N2 Bristol) worms. Representative raw traces from Clarke electrode (E), and tabulated data normalized by worm number (F).

As part of its normal metabolism, *C. elegans* generates an assortment of metabolic end products that move from its tissues to the external environment (collectively defining the worm exometabolome). Using gas chromatography–mass spectrometry (GC–MS), we detected some 200 metabolic components within the exometabolome of wild‐type worms (Butler *et al*., 2012). When we quantified the exometabolome of four different Mit mutants [*clk‐1(qm30)*,* isp‐1(qm150)*,* nuo‐6(qm200)*, and *tpk‐1(qm162)*], we made the surprising finding that all were uniquely enriched with an ensemble of α‐hydroxyacids and α‐ketoacids (Fig. 1A)—compounds that were not detected in other long‐lived mutants, including *daf‐2(e1370)*,* eat‐2(ad465)*,* clk‐2(qm37)*, and *sclf‐1(tm2285)* (Butler *et al*., 2012). Intriguingly, neither were these compounds found enriched in mutants that had disrupted mitochondrial electron transport chains but which were short‐lived [*mev‐1(kn1)* and *ucr‐2.3(pk732)*]. Our most recent evidence suggests that these chemicals may arise following functional impairment of three closely related α‐ketoacid dehydrogenase complexes: branched‐chain α‐ketoacid dehydrogenase (BCKADH), α‐ketoglutarate dehydrogenase (α‐KGDH), and pyruvate dehydrogenase (PDH) (Butler *et al*., 2013). The ensemble of α‐hydroxyacids and α‐ketoacids in Mit mutants is of particular interest because these compounds share structural similarity with α‐ketoglutarate, a metabolite recently shown to bind the β‐subunit (ATP‐2) of the mitochondrial F_1_F_o_ATP synthase (complex V) and to extend nematode lifespan (Chin *et al*., 2014). Binding by α‐ketoglutarate reduced complex V activity, decreased oxygen consumption, and increased autophagy. α‐ketoglutarate is also a required co‐substrate for an evolutionarily conserved class of iron‐ and oxygen‐dependent hydroxylases and N‐demethylases called the α‐ketoglutarate‐dependent hydroxylases (Fig. 1B). This diverse family of cupin‐fold containing enzymes carries out a range of functions including epigenetic regulation, DNA damage repair, tRNA‐modification, axonal regeneration, and metabolite processing (McDonough *et al*., 2010; Neumann *et al*., 2015). Such findings raise the possibility that the α‐hydroxyacids and α‐ketoacids found in Mit mutants could play a role in establishing their phenotype.

Several α‐ketoglutarate‐dependent hydroxylases have been linked with life extension in worms (Maures *et al*. 2011; Ni *et al*., 2012), including the prolyl‐hydroxylase EGL‐9 (Lee *et al*., 2010), which regulates the stability of hypoxia‐inducible factor‐1 (HIF‐1). HIF‐1 is a dimeric protein composed of α‐ and β‐subunits and in multiple species functions as a master transcription factor that coordinates survival during hypoxic conditions. Intriguingly, HIF‐1 activation is required for the life extension of Mit mutants, regardless of oxygen level; that is, both genetic and RNAi‐mediated knockdown of HIF‐1α have been shown to abolish Mit mutant longevity even under normoxic conditions (Lee *et al*., 2010; Khan *et al*., 2013). The mode of HIF‐1 activation in these animals, however, has yet to be determined. In mammalian cancer cells, it is known that the PI3‐kinase/AKT signaling axis can lead to elevated HIF‐1α protein levels under normoxic conditions and that this pathway is sensitive to antioxidant administration (Liu *et al*., 2011; Kilic‐Eren *et al*., 2013). This is interesting because treatment of *isp‐1(qm150)* and *nuo‐6(qm200)* Mit mutants with N‐acetyl cysteine totally suppresses their life extension (Yang & Hekimi, 2010a). The tumor suppressor PTEN, a negative regulator of AKT signaling, can be rendered catalytically inactive through oxidation by reactive oxygen species (ROS) (Chetram *et al*., 2013), providing a potential link between ROS, PI3‐kinase signaling, and HIF‐1 activation under normoxia. The PI3‐kinase/AGE‐1 signaling axis, however, does not appear to be involved in Mit mutant life extension (Feng *et al*., 2001), implying another mechanism leading to HIF activation must be in play. A novel life‐extending signal involving ROS, CED‐13, and the intrinsic apoptotic pathway was recently delineated in *isp‐1(qm150)* and *nuo‐6(qm200)* Mit mutants (Yee *et al*., 2014), but this pathway also seems to operate independently of HIF‐1α (Yang & Hekimi, 2010a).

How then does HIF‐1 become activated in Mit mutants? Potential insight again comes from cancer cell studies: ROS can stabilize HIF‐1α in an AKT‐independent manner and this effect is mediated by the oxidative consumption of ascorbate and Fe(II) (Li *et al*., 2014). Under normal circumstances, these two compounds are utilized by the EGL‐9/PHD1‐3 prolyl‐hydroxylases to post‐translationally hydroxylate HIF‐1α and in turn permit its recognition by the E3 ubiquitin ligase VHL‐1, leading to its subsequent degradation by the proteasome. Very recently, it was shown that low iron levels stabilize HIF‐1α in *frh‐1* mutants of *C. elegans*, which are defective for frataxin expression and share many phenotypes with Mit mutants (Schiavi *et al*., 2015). Another possibility for activating HIF‐1 involves endogenous, α‐ketoglutarate‐like compounds that have been collectively described as ‘oncometabolites’. These compounds are made within cells as part of normal intermediary metabolism but, when present at supranormal levels due to some underlying pathology, promote tumorigenesis. Four such oncometabolites (L‐2‐hydroxyglutarate, D‐2‐hydroxyglutarate, succinate and fumarate) are known to competitively target the active site of several α‐ketoglutarate‐dependent hydroxylases—including EGL‐9/PHD1‐3 and jumonji domain‐containing (JmjC) histone demethylases (JMJDs), and of these proteins some are known to regulate lifespan in *C. elegans* (Xu *et al*., 2011).

Having found that multiple α‐ketoglutarate analogues accumulate in the exometabolome of Mit mutants, we have tested here whether any are causally involved in generating the long‐lived Mit phenotype.

## Results

The exometabolome of genetically defined Mit mutants is enriched in several compounds that are normally metabolized by mitochondrial α‐ketoacid dehydrogenase complexes, including α‐ketobutyrate (2‐oxobutyrate, 2OB), α‐ketoisocaproate (4‐methyl‐2‐oxovalerate, 4M2OV), α‐ketoisovalerate (3‐methyl‐2‐oxobutyrate, 3M2OB), α‐keto‐β‐methylvalerate (3‐methyl‐2‐oxovalerate, 3M2OV), and α‐ketopropionate (pyruvate) ((Butler *et al*., 2013) and Fig. 1C). Increased amounts of the reduced, α‐hydroxyacid form of these α‐ketoacids are also observed. These pairs of compounds exist in a dehydrogenase‐catalyzed redox equilibrium with NAD (Williamson *et al*., 1967). The α‐ketoacid dehydrogenase complexes are comprised of multiple E1, E2, and E3 subunits that assemble into a mini‐electron transport chain which normally operates sequentially in the order of α‐ketoacid oxidation (E1 subunit), lipoic acid reduction (E2 subunit), cystine reduction, FAD reduction, and NAD+ reduction (the three latter sites all reside within the E3 subunit). Several studies have shown that the α‐ketoacid dehydrogenase complexes can become sites of massive ROS generation when electrons are reverse‐fed into the complexes by elevated NADH levels (Bryk *et al*., 2002; Gazaryan *et al*., 2002; Argyrou *et al*., 2003; Starkov *et al*., 2004). This is because α‐ketoacid oxidation catalyzed by E1 is an irreversible reaction and so electrons become lodged on the highly reactive lipoic acid that is covalently attached to E2. Based on these findings, we have previously speculated that hindered flow of electrons through the mitochondrial electron transport chain in Mit mutants leads to a build‐up of matrix NADH, reverse electron flow through the α‐ketoacid dehydrogenases, and ultimately α‐ketoacid accumulation (Butler *et al*., 2013). The *isp‐1(qm150)* Mit mutant allele encodes a missense point mutation in the Rieske Fe–S protein of complex III (Feng *et al*., 2001) that manifests phenotypically as a complex I defect due to ineffective supercomplex formation (Suthammarak *et al*., 2010). In support of our previous hypothesis, we now demonstrate that forced ETC consumption of NADH in *isp‐1(qm150)* Mit mutants, following addition of the mitochondrial uncoupler FCCP, is sufficient to mitigate accumulation of α‐ketoacids in their exometabolome (Fig. 1D). When applied acutely, FCCP collapses the mitochondrial membrane potential (Δψ_m_) and stimulates cellular respiration (Si *et al*., 2009). *isp‐1(qm150)* Mit mutants respond to FCCP treatment within seconds (Fig. 1E) in a manner indistinguishable from wild‐type (N2 Bristol) worms (Fig. 1F), illustrating that collapse of Δψ_m_ triggers a compensatory increase in ETC flux that occurs independently of the hypomorphic *isp‐1(qm150)* allele—possibly by stimulating supercomplex assembly (Feng *et al*., 2001; Suthammarak *et al*., 2010).

### Mit mutant exometabolites extend the lifespan of wild‐type worms

**Figure 2:**
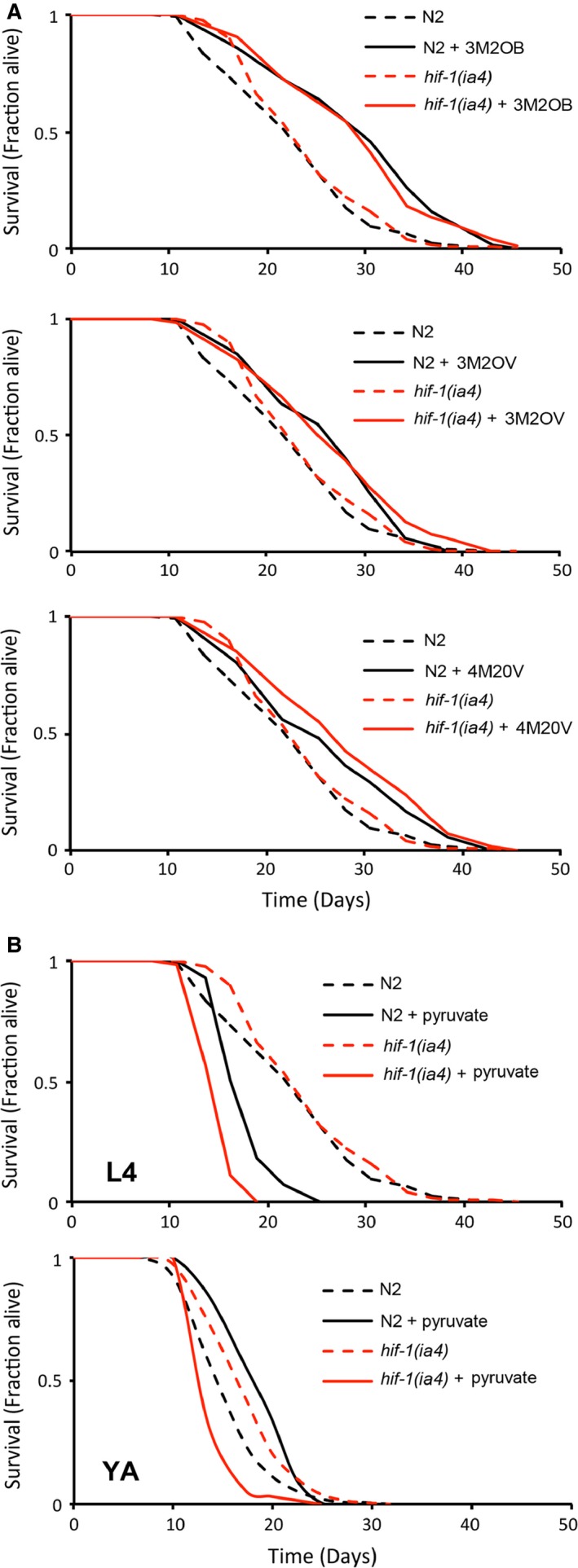
Mit mutant exometabolites extend the lifespan of wild‐type worms when provided as dietary supplements. (A, B) Adult lifespan of wild‐type (N2) and mutant *hif‐1(ia4)* worms following dietary supplementation with 10 mm 3M2OB, 3M2OV, 4M2OV, or 8 mm pyruvate from the L4 larval stage of development onwards. All three BCKAs significantly extended lifespan in both genetic backgrounds (*P* < 0.05, Log‐rank test). Pyruvate significantly extended the lifespan of wild‐type worms only when added at the young adult (YA) stage and only in the presence of functional *hif‐1* (B).

**Figure 3:**
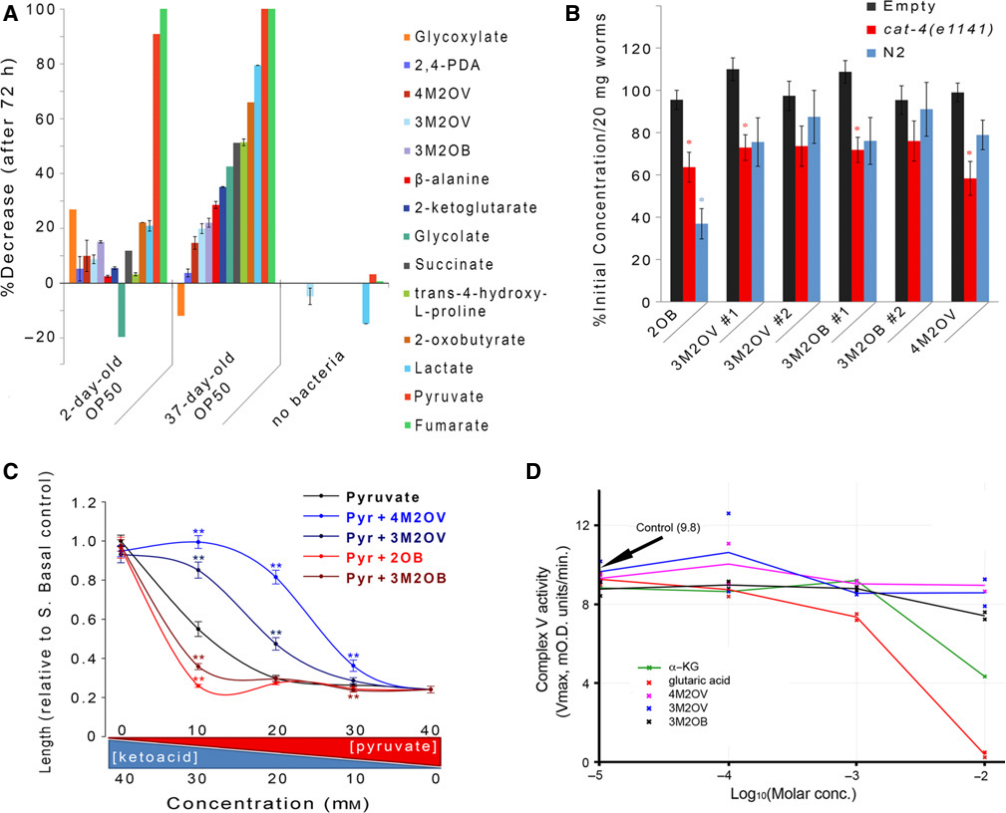
Exometabolite pharmacodynamics. (A) Stability of α‐ketoacid test compounds in the presence of bacteria. OP50 bacteria were incubated in minimal media supplemented with one of the listed compounds for 72 h [2,4‐pyridinedicarboxylic acid (2,4‐PDA)]. Chemical that remained after this time was quantified by ^1^H NMR. Shown is the average change in ^1^H signal relative to time zero, and which, for each chemical, was uniquely calculated by averaging over all types of proton chemical shifts (‘error bars’ represent ± SEM for compounds with >1 chemical shift, see also Table S2). The effect of bacterial culture age on each chemical's stability was also tested (2‐day vs. 37‐day bacteria stored at 4 °C prior to use). Not all chemicals were tested in the absence of bacteria. (B) Assimilation of branched‐chain α‐ketoacids by wild‐type (N2) and *cat‐4(e1141)* worms. Animals were cultured for 18 h in liquid medium containing the listed compound. Compound depletion was determined by GC‐MS. Evaporative losses were controlled using a sample devoid of animals (*empty)*. Bars represent combined mean ± SE (*n* = 9 for both N2 worms and evaporative control, *n* = 7 for *cat‐4* worms). Asterisks indicate significant difference from no worm control (*P* < 0.05, Student's *t*‐test with Holm–Bonferroni correction for multiple testing; 2‐oxobutyrate, 2OB). (C) 3M2OV and 4M2OV compete with pyruvate for transport into worms. *cat‐4(e1141)* worms were treated with various concentrations of pyruvate + test α‐ketoacid for 72 h. from the time of hatching. When added to L1 larvae, pyruvate causes a concentration‐dependent slowing of development (Fig. S12), but in the presence of 3M2OV or 4M2OV development re‐attains wild‐type rates. Animal length was used as a continuous measure for larval stage. For each data point, the combined chemical concentration (pyruvate + α‐ketoacid) was 40 mm. At least ten animals were measured for each condition tested. (Error bars: ±1 SD, *^*^ *P* < 0.05). (D) Effect of 3M2OB, 3M2OV, 4M2OV, α‐ketoglutarate, and glutarate on the activity of mitochondrial complex V isolated from bovine heart mitochondria. Lines represent averages of duplicate titration experiments, except α‐ketoglutarate which is a single titration experiment. Average *V*_max_ flux in the absence of test chemicals is marked (arrow).

To determine whether compounds that accumulate in the exometabolome of Mit mutants could conceivably play a role in specifying the extended life of these animals, we exposed wild‐type worms to several test compounds and measured their survival (Table S1). We tested a range of concentrations, under a variety of culture conditions, starting at multiple stages of larval development. The three branched‐chain α‐ketoacids (BCKAs)—3M2OB, 3M2OV, and 4M2OV derived from valine, isoleucine, and leucine transamination, respectively, reproducibly and significantly (*P* < 0.01) increased lifespan when fed to wild‐type worms from the L4 stage of larval development (Fig. 2A, *black lines* and Table S1). A fourth α‐ketoacid, pyruvate, also increased the lifespan of wild‐type worms, but surprisingly only when supplemented from the young adult stage of development (Fig. 2B, *black lines* and Table S1). In all four instances, worms were incubated with 10 mm of the test compound in the presence of 50 μm 5‐fluorodeoxyuridine (FuDR), to sterilize animals. This test dose is comparable to the concentration of α‐ketoglutarte previously found to extend life in worms (Chin *et al*., 2014). Although 1–2 orders of magnitude greater than most intermediary metabolites (Dienel, 2009), the effective delivery dose for each compound was likely much less than 10 mm for the following reasons. In our hands, use of heat‐killed *E. coli* (OP50) did not support worm development and so live bacteria were utilized. Under these assay conditions, pyruvate was readily metabolized by OP50, as revealed using ^1^H NMR analysis (Fig. 3A). 3M2OB, 3M2OV, and 4M2OV were also metabolized by bacteria, but to a lesser extent (Figs 3A,B, S2–S9, Table S2). Furthermore, wild‐type worms incorporate 3M2OV and 4M2OV with low efficiency because these compounds are easily out competed by smaller α‐ketoacids for their uptake (Fig. 3C). Nonetheless, our findings indicate that compounds that are aberrantly generated in Mit mutants play a role in controlling worm longevity.

### Weak complex V inhibition by 3M2OB

We next sought to determine how pyruvate and the three branched‐chain α‐ketoacids act to increase lifespan in wild‐type worms. α‐ketoglutarate was recently shown to bind and inhibit the β‐subunit (ATP‐2) of the mitochondrial F_1_F_o_ATP synthase (complex V) in both worm and bovine heart mitochondrial samples, and to extend nematode lifespan (Chin *et al*., 2014). Using the same drug affinity responsive target stability (DARTS) assay originally used to identify α‐ketoglutarate binding to ATP‐2, we found no evidence that 3M2OB, 3M2OV, 4M2OV, or pyruvate could bind ATP‐2 in whole‐worm lysates (data not shown). Utilizing a more sensitive enzyme‐linked immunosorbent (EIA) assay, in conjunction with purified bovine heart mitochondria, 3M2OB was found to inhibit complex V inhibition, albeit weakly and only at the highest tested dose (10 mm) (Fig. 3D). Pyruvate was a substrate in the coupled assay, which precluded its testing (Data S1). Interestingly, during the course of our studies, we discovered that glutarate is a much more potent complex V inhibitor than α‐ketoglutarate (Fig. 3D), suggesting this compound might be the biologically relevant inhibitor (refer to Discussion).

### Mit mutant exometabolites inhibit the histone demethylase JMJD2A/JMJD‐2, *in vitro*, but do not cause gross histone methylation alterations in worms

**Figure 4:**
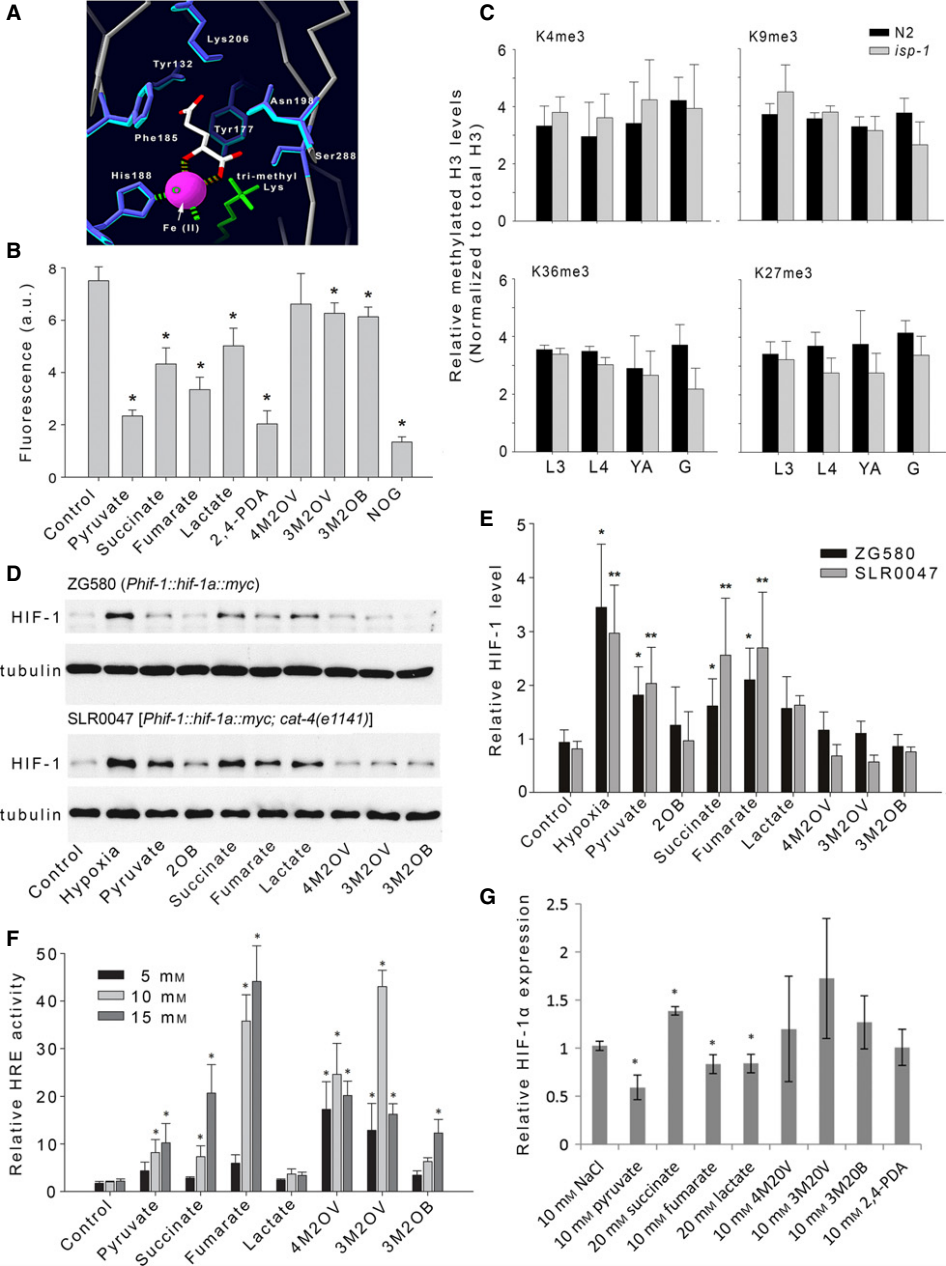
Mit mutant metabolites increase HIF‐1 levels in worms and HIF transcriptional activity in 3T3‐L1 fibroblasts. (A) Homology model of JMJD‐2. Shown is the active site of both JMJD‐2 and JMJD2A illustrating their structural conservation. Only residues within 6.2 Å of the C4 carbon of the co‐crystallized R‐2‐hydroxyglutarate inhibitor (white) are shown. Amino acid side chains in JMJD‐2 (*dark blue*) predicted to occupy the same topological space as active site side chains of JMJD2A (*light blue*) are illustrated. Ni/Fe(II)—*pink ball*; H3K36me3 (αα 30‐41)—*green*. (Numbering in panel refers to JMJD2A amino acids). (B) Recombinant JMJD2A protein was incubated with its prime substrate (H3K9me3 peptide), α‐ketoglutarate (0.5 mm) and the indicated compound (10 mm). Activity was measured by following formaldehyde production using a fluorescence assay (presented as relative absorbance units). Data is normalized to enzymatic activity in the absence of any test metabolite (*control)*. Bars represent mean (±SEM) of duplicate independent experiments, assayed in triplicate. Asterisks indicate significantly difference with respect to control (*P* < 0.05, anova, Holm–Sidak *post hoc* analysis). 2‐oxobutyrate (2OB); 4‐methyl‐2‐oxovalerate (4M2OV); 3‐methyl‐2‐oxovalerate (3M2OV); 3‐methyl‐2‐oxobutyrate (3M2OB); N‐oxalylglycine (NOG); 2,4‐pyridinedicarboxylic acid (2,4‐PDA). (C) Comparison of histone H3 methylation levels in wild‐type (N2) animals versus *isp‐1(qm150)* Mit mutants. Histone H3 modifications (listed) were quantified by Western analysis. Bar plots represent mean data from three independent experiments (±1 SD). Four developmental stages were analyzed—L3 and L4 larvae, young adults (YA) and gravid adults (G). No significant differences were observed between strains (*P* >* 0*.05, anova). (D) Representative Western analysis showing acute effects of the listed compounds (40 mm each) on expression of a transgenic HIF‐1::myc reporter in otherwise wild‐type animals (ZG580) and *cat‐4(e1141)* mutants with enhanced cuticle permeability (SLR0047). Both untreated and positive (hypoxia‐exposed) controls are included. Test strain and genotype are indicated. (E) Quantification of all HIF‐1::*myc* Western analyses described in (D). Data has been normalized to untreated control worms. Bars represent mean (±1 SEM) of three independent experiments per strain. Asterisks indicate significant difference with respect to untreated control (*P* < 0.05, *t*‐test). (F) 3T3‐L1 fibroblasts containing a luciferase reporter under the control of a HIF response element (3T3‐L1‐HRE‐*LucD)* were treated with the indicated metabolite (at 5, 10, and 15 mm) for 20 h. Bars represent average HRE response activity from three independent experiments (error bars: ±1 SD). Data has been normalized to a NaCl‐matched control. Asterisks indicate significant difference with respect to control (*P* < 0.05, *t*‐test). (G) Quantification of HIF‐1α mRNA levels in 3T3‐L1‐HRE‐*LucD* cells at the end of the treatments in (F). Only the listed concentration was analyzed. Data represents average of three independent experiments (error bars: ±1 SD) and is normalized to the NaCl‐matched control. Asterisks indicate significant difference with respect to control (*P* < 0.05, *t*‐test).

Given the structural similarity between 3M2OB, 3M2OV, 4M2OV, pyruvate, and α‐ketoglutarate, we next focused our attention on the α‐ketoglutarate‐dependent hydroxylases which use the latter compound as an obligatory co‐substrate (Fig. 1B,C). Also, pyruvate is known to inhibit two members of this enzyme family in other species (Table S3). Among the α‐ketoglutarate‐dependent hydroxylases, there are two broad classes that differ by the ensemble of residues that have evolved in their active site to chelate iron and α‐ketoglutarate, both of which are necessary for hydroxylase activity (McDonough *et al*., 2010). The HIF‐1 prolyl‐hydroxylases and the jumonji domain C‐terminal (JmjC)‐type histone demethylases are prototypical members of the two respective classes. Previous studies have shown that RNAi‐mediated inhibition of the gene encoding the JmjC histone demethylase JMJD‐2 in wild‐type worms is sufficient to increase lifespan (Ni *et al*., 2012). JMJD‐2 is orthologous to human JMJD2A (Whetstine *et al*., 2006) and homology modeling reveals that both proteins have active site cavities that are 100% conserved (Fig. 4A). Using a commercially available recombinant JMJD2A, we first assessed whether 3M2OB, 3M2OV, 4M2OV, or pyruvate could inhibit the histone demethylase activity of this protein. As shown in Fig. 4B, pyruvate, and less so 3M2OB and 3M2OV, reproducibly and significantly (*P* < 0.05) inhibited JMJD2A activity. Succinate (a by‐product of the hydroxylation reaction), lactate (the α‐hydroxyacid form of pyruvate and which also accumulates in the exometabolome of Mit mutants), as well as two α‐ketoglutarate mimetics—2,4‐pyridinedicarboxylic acid (2,4‐PDA) and the α‐ketoacid N‐oxalylglycine (NOG), all also significantly blocked JMJD2A activity. Among the α‐ketoacids, pyruvate (the smallest of the group) had the greatest inhibitory effect—reducing JMJD2A activity by 85%. The largest α‐ketoacid (4M2OV) was the only compound without effect, strongly suggesting steric hindrance limited its entrance into the active site. To determine whether histone methylation patterns were altered *in vivo*, we next used Western analysis to quantify histone H3 methylation levels in both *isp‐1(qm150)* Mit mutants and wild‐type worms. Relevant targets of JmjC histone demethylases (Loenarz & Schofield, 2008) were measured in whole‐worm extracts across multiple stages of development, and included H3K4me3, H3K27me3, H3K9me3, and H3K36me3. The latter three sites are demethylated by JMJD‐2/JMJD2A (Williams *et al*., 2014). Analysis at this gross level, however, revealed no significant disruption in the methylation patterns of these four targets (Fig. 4E). These data suggest that JmjC histone demethylases are not targeted to any great extent by α‐ketoacids *in vivo*, or, if they are, that they are below the resolving power of our assay.

### Mit mutant exometabolites stabilize HIF‐1 protein levels in wild‐type *C. elegans*

In wild‐type worms, HIF‐1 (the worm ortholog of HIF‐1α) is stabilized under hypoxic conditions because its negative regulator EGL‐9/PHD1‐3 requires oxygen for its catalytic cycle (Epstein *et al*., 2001). Like other α‐ketoglutarate‐dependent hydroxylases, EGL‐9 consumes α‐ketoglutarate during its catalytic cycle (Dowell & Hadley, 1992). Surprisingly, HIF‐1 is constitutively active under normoxic conditions in Mit mutants (Lee *et al*., 2010; Khan *et al*., 2013), suggesting EGL‐9 could be a target of 3M2OB, 3M2OV, 4M2OV, and/or pyruvate in these animals. We tested whether supplementation of these four α‐ketoacids could stabilize a functional HIF‐1::*myc* translational reporter when provided to 1‐day‐old adult worms. Four other compounds that are either structurally or functionally related to α‐ketoglutarate were also included in this assay—2‐OB, lactate, succinate, and fumarate (Fig. 1B,C). As a positive control, a sample of HIF‐1::*myc* worms was exposed to hypoxia. When HIF‐1::*myc* protein levels were quantified by Western analysis, we found three compounds—pyruvate, succinate, and fumarate, consistently and significantly (*P* < 0.05), increased HIF‐1::*myc* above the level in untreated control worms, across triplicate independent experiments (Fig. 4D, *top panel* and E). Lactate also trended toward significance (*P* < 0.13). We next crossed our HIF‐1::*myc* reporter into *cat‐4(e1141)* mutant worms, which have increased cuticular permeability (see Supporting Information), and then, we repeated our analysis (Fig. 4D, *lower panel* and E). Using this genetic background, identical results were observed. These data illustrate that at least one compound, pyruvate, that accumulates in the exometabolome of Mit mutants and is sufficient to increase the lifespan of wild‐type worms (Fig. 2B, lower panel, black lines), is also sufficient to stabilize a factor known to be essential for Mit mutant longevity, namely HIF‐1 (Lee *et al*., 2010; Khan *et al*., 2013). Indeed, the pro‐longevity effect of pyruvate on wild‐type worms is abrogated following disruption of *hif‐1* (Fig. 2B, lower panel, red lines). *hif‐1* is not required, however, for the life‐promoting effects of 3M2OB, 3M2OV, or 4M2OV (Fig. 2A, red lines), suggesting these compounds function in a manner distinct from pyruvate.

### Mit mutant exometabolites enhance HIF‐1 transcriptional activity when added to mouse 3T3‐L1 fibroblasts

We next asked whether the effects we observed on HIF‐1 protein level in *C. elegans* following administration of individual α‐hydroxyacids and α‐ketoacids could be replicated in mammalian cells. We generated a mouse 3T3‐L1 fibroblast reporter line (3T3‐L1‐HRE‐*LucD*) that stably expressed a previously characterized, firefly luciferase reporter under the control of a HIF‐1 response element (HRE) (Fishel *et al*., 2011). This line also expressed a constitutively active *Renilla* luciferase reporter gene to serve as an internal normalization control. This cell line allowed us to monitor the transcriptional competency of HIF‐1. Consistent with our findings in worms, where addition of pyruvate, succinate, or fumarate stabilized HIF‐1::*myc* protein, addition of these same compounds to 3T3‐L1‐HRE‐*LucD* cells increased the transcriptional activity of HIF‐1 (Fig. 4F). Moreover, the three branched‐chain α‐ketoacids, 3M2OB, 3M2OV, 4M2OV, which had only a marginal effect on HIF‐1::*myc* protein levels in worms, significantly enhanced the transcriptional activity of HIF‐1 in 3T3‐L1 cells. The three tested doses presented in Fig. 4F illustrate that each metabolite had an optimal concentration that invoked maximal HRE activity. For two of the metabolites, 3M2OV and 4M2OV, there was a decrease in HRE activity at the highest dose (15 mm) relative to their respective optimal dose, likely due to reduced cell viability (Data S1). We excluded the possibility that the effect of each metabolite on HIF‐1 transcriptional activity was due simply to an increase in HIF‐1α transcript abundance. qRT–PCR analysis revealed that pyruvate, fumarate, and lactate caused a significant (*P* < 0.05) *decrease* in HIF‐1α mRNA, while the three branched‐chain α‐ketoacids caused no significant change (Fig. 4G). For succinate, we observed a slight increase in HIF‐1α mRNA, although this was still an order of magnitude lower than the observed degree of HRE activation. These data illustrate that compounds found in the Mit exometabolome can lead to hyperactivation of a HIF‐1 reporter gene in the context of mammalian cells and that HIF‐1 stabilization following addition of these metabolites occurs at a posttranslational level, likely by EGL‐9/PHD inhibition.

### 2,4‐pyridinedicarboxylic acid (2,4‐PDA) extends the lifespan of wild‐type worms in a *hif‐1*‐dependent manner

**Figure 5:**
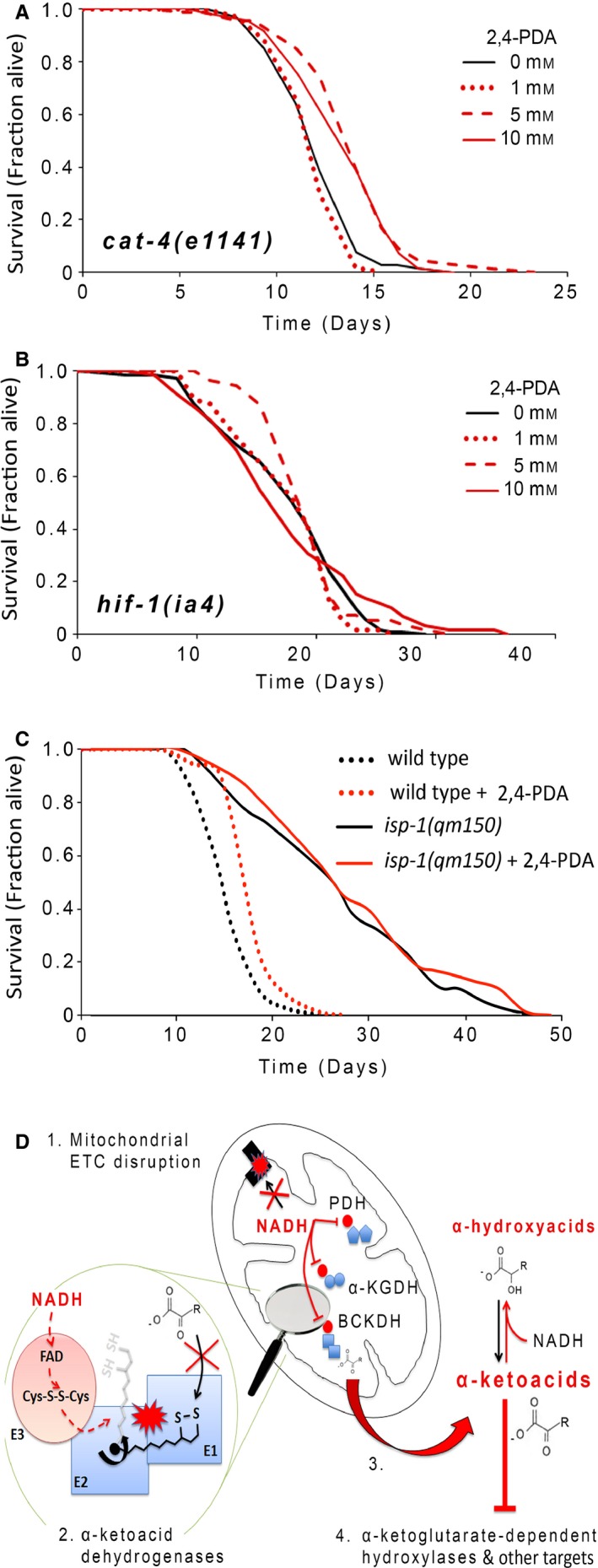
2,4‐PDA extends nematode survival in a HIF‐1‐dependent manner, but does not further increase the lifespan of *isp‐1(qm150)* Mit mutants. (A) *cat‐4(e1141)* worms were supplemented from the time of hatching with 1, 5, or 10 mm 2,4‐PDA and adult survival analyzed relative to untreated animals. Both 5 and 10 mm 2,4‐PDA significantly extended lifespan (*P* < 0.05, Log‐rank test). (B) *hif‐1(ia4)* mutants fail to show life extension when supplemented with 5 and 10 mm 2,4‐PDA. (C) The life‐extending effect of 5 mm 2,4‐PDA is not additive with that induced by the *isp‐1(qm150)* mutation. Descriptive statistics for all survival analyses are provided in Table S1. (D) Model for cause and effects of α‐ketoacid generation in Mit mutants. (1) ETC dysfunction increases NADH levels in mitochondrial matrix. (2) Electrons from NADH reverse flow into α‐ketoacid dehydrogenases (PDH, BCKADH, α‐KGDH) resulting in their inactivation. (3) α‐ketoacids accumulate outside mitochondria, along with cognate α‐hydroxyacids. (4) Pyruvate targets EGL‐9 leading to stabilization of HIF‐1, causing life extension. Other α‐ketoacids may competitively target additional α‐ketoglutarate‐dependent hydroxylases, or other cellular components (e.g., 3M2OB and complex V) to increase lifespan.

Identification of compounds that mimic the life‐extending effects of worm metabolites, and which are stable to bacterial degradation, would provide a useful tool for further studies in this and other organisms. As mentioned earlier, 2,4‐PDA is an α‐ketoglutarate mimetic that inhibits multiple α‐ketoglutarate‐dependent hydroxylases (Rose *et al*., 2011) (Table S3). 2,4‐PDA binds to the active site of these enzymes with low micromolar affinity, and competitively with respect to α‐ketoglutarate (Majamaa *et al*., 1984). In humans, several α‐ketoglutarate‐dependent hydroxylases are targeted by 2,4‐PDA (Hopkinson *et al*., 2013); among these is the HIF prolyl‐hydroxylase PHD2, as well as multiple JmjC histone demethylases—including JMJD2A (see also Fig. 4B). We now show that 2,4‐PDA is both resistant to degradation by OP50 bacteria (both live and dead)—as revealed using ^1^H NMR analysis (Figs 3A, S9 and Table S4), and dietary supplementation reproducibly extends the adult lifespan of *C. elegans* by up to 15%. This effect was robust across three different genetic backgrounds—wild‐type worms (N2 Bristol), transgenic worms containing an oxidative stress reporter (*gst‐4::GFP)*, and *cat‐4(e1141)* mutants (Fig. 5A,C and Table S1). If EGL‐9 is a major target of 2,4‐PDA in worms, then we would predict that a *hif‐1* loss‐of‐function mutation should abrogate the life‐extending effects of this drug. Indeed, we find that *hif‐1(ia4)* mutants fail to exhibit life extension when fed 2,4‐PDA (Fig. 5B). Moreover, when administered to HIF‐1::*myc* reporter worms, 2,4‐PDA stabilized HIF‐1::*myc* expression (Fig. S10). Together, these data suggest that an EGL‐9/HIF‐1 signaling axis mediates the life‐extending effects of 2,4‐PDA in worms.

### No additional life extension in *isp‐1(qm150)* Mit mutants treated with 2,4‐PDA

Among the four α‐ketoacids identified from the exometabolism of Mit mutants that increase lifespan when supplemented to wild‐type worms, only pyruvate caused robust stabilization of HIF‐1 (Fig. 4D,E). We and others have shown that HIF‐1 is absolutely essential for the life extension of Mit mutants (Lee *et al*., 2010; Khan *et al*., 2013). If pyruvate works to stabilize HIF‐1 by competitively inhibiting the active site of EGL‐9, then we would predict that administration of 2,4‐PDA to *isp‐1(qm150)* Mit mutants should not further extend the lifespan of these animals. This is indeed the case—exposure of *isp‐1(qm150)* mutants to 2,4‐PDA throughout their life resulted in no additional extension of lifespan, but did increase the lifespan of wild‐type control animals (Fig. 5C). These data are consistent with the notion that the aberrant accumulation of pyruvate by *isp‐1(qm150)* Mit mutants is not benign but almost certainly has a functional consequence inside cells where it competitively inhibits EGL‐9, leading to HIF‐1 stabilization and ultimately life extension (see model, Fig. 5D).

## Discussion

In this study, we tested the hypothesis that compounds that accumulate in the exometabolome of long‐lived Mit mutants play a direct role in the lifespan specification of these animals. We observed that dietary supplementation of four compounds to wild‐type worms, namely pyruvate and the three branched‐chain α‐ketoacids—3M2OB, 3M2OV, and 4M2OV, was sufficient to extend life, and in the case of pyruvate, we showed that this effect was mediated by stabilization of HIF‐1, a transcription factor previously implicated in the longevity of Mit mutants (Lee *et al*., 2010; Khan *et al*., 2013; Schiavi *et al*., 2015). We also found evidence that 3M2OB weakly inhibited mitochondrial complex V activity. This is of interest because the structurally related compound α‐ketoglutarate was recently shown to inhibit complex V, and to increase autophagy and extend the life of wild‐type worms (Chin *et al*., 2014). α‐ketoglutarate was also shown in the same study to inhibit complex V in mouse and human cell lines and to reduce target of rapamycin (TOR) activity. Intriguingly, in the present study, we discovered that glutarate was a more potent complex V inhibitor than α‐ketoglutarate—suggesting the former compound might be the biologically relevant inhibitor. Glutaryl‐CoA, and subsequently glutarate, is generated during the α‐ketoglutarate dehydrogenase‐catalyzed oxidation of 2‐oxoadipate, which itself is generated during lysine catabolism. It is unclear why glutarate would evolve to inhibit complex V activity, but one possibility is that if glutarate levels accumulate during severe starvation when fat supplies are exhausted, it might act to limit cachexia. Inhibition of complex V by glutarate has not been previously described, but it may help explain the mitochondrial pathology that accompanies glutaric acidemias (Hedlund *et al*., 2006; Ferreira *et al*., 2007).

Solari and colleagues previously reported that pyruvate supplementation could extend the lifespan of wild‐type worms (Mouchiroud *et al*., 2011). This work was undertaken in the context of characterization of a mutant monocarboxylate transport protein, SLCF‐1, which at the time was a novel longevity factor in *C. elegans*. These authors showed that mutant *slcf‐1(tm2258)* worms were retarded in their capacity to export pyruvate and that pyruvate accumulation was responsible for life extension. They showed that treatment of *slcf‐1(tm2258)* worms with N‐acetylcysteine (NAC) abrogated longevity, and similarly genetic removal of PTEN/daf‐18, AMPK/*aak‐2*, FOXA/*pha‐4, SIRT/sir‐2.1*, and HSF/*hsf‐1* each blocked the life extension conferred by loss of *slcf‐1*. These findings are consistent with our current work where we now show that pyruvate triggers life extension in wild‐type worms in a HIF‐1‐dependent manner. We speculate that NAC abrogated the life‐prolonging effects of the *slcf‐1(tm2258)* mutation in the studies of Solari and colleagues (Mouchiroud *et al*., 2011) because it functioned either alongside or in conjunction with ascorbate—another antioxidant, which is required to keep the active site of EGL‐9 and other α‐ketoglutarate‐dependent hydroxylases in the Fe(II) state (Myllyharju, 2008). Finally, Solari and colleagues (Mouchiroud *et al*., 2011) also showed the life extension following *slcf‐1* RNAi did not further extend the lifespan of *isp‐1(qm150)* Mit mutants. Again, this observation is consistent with our current findings where we show that treatment of *isp‐1(qm150)* worms with 2,4‐PDA, which mimics pyruvate in both its stabilization of HIF‐1 and its ability to extend the life of wild‐type worms, did not further extend the lifespan of these mutants.

Presently, it remains unclear how 3M2OV and 4M2OV function to extend lifespan. Given their structural similarity to 3M2OB, we think it is probable that all three compounds will work through the same target(s), and for this reason, we think it is unlikely that inhibition of complex V is mediating their pro‐longevity effects. In this regard, although we show that 3M2OB could weakly inhibit complex V in bovine heart mitochondrial extracts, we were unable to detect an interaction between ATP‐2 and any of the three branched‐chain α‐ketoacids using the same DARTS approach that was originally used to identify ATP‐2 as a target of α‐ketoglutarate in worms (Chin *et al*., 2014). Currently, we favor the hypothesis that 3M2OB, 3M2OV, and 4M2OV target another member of the α‐ketoglutarate‐dependent hydroxylase family that is not targeted by 2,4‐PDA (Hopkinson *et al*., 2013). As shown in Fig. 1B, this large family of enzymes requires multiple co‐substrates for activity. The catalytic mechanism involves α‐ketoglutarate first binding to active site Fe(II) using both its C‐1 carboxylate and C‐2 keto groups. Next, each enzyme has its own unique target, or set of targets, and entry of this ‘prime’ substrate into the active site is thought to weaken an H_2_O‐Fe(II) bond, allowing dioxygen to bind Fe(II). Subsequent oxidative decomposition of α‐ketoglutarate results in release of both CO_2_ and succinate, as well as generation of a strongly oxidizing Fe(IV)‐oxo intermediate that ultimately hydroxylates the prime substrate (McDonough *et al*., 2010). It is the large, cupin barrel structure which houses the active site of these enzymes that also permits various α‐hydroxyacids and α‐ketoacids to enter and competitively bind Fe(II) with respect to α‐ketoglutarate. In *C. elegans,* there are 48 α‐ketoglutarate‐dependent hydroxylases (Finn *et al*., 2010), at least four of which have been shown to regulate survival when inhibited in wild‐type worms: EGL‐9 which, as we have discussed, negatively regulates HIF‐1 (Lee *et al*., 2010); the H3K27 JmjC histone demethylase UTX‐1 (Maures *et al*., 2011); and two other JmjC histone demethylases, JMJD‐2 and RBR‐2 (Ni *et al*., 2012). Despite this, we observed no changes in the histone methylation patterns of *isp‐1(qm150)* worms indicative of JmjC‐type inhibition. One limitation of our analysis was that we used whole‐worm extracts—meaning that *bona fide* changes in histone methylation patterns of only a subset a cells would have been likely to have gone undetected. We simply may have missed such changes. Moreover, we only tested four of the more than 30 methyl‐histone regulatory changes that are known (although not all are controlled by JmjC‐type demethylases), and so we cannot yet definitively rule out a role for α‐ketoacids in the epigenetic regulation of Mit mutant lifespan.

In the current study, we also showed that murine 3T3‐L1 cells containing a luciferase reporter gene under the control of a HIF‐1 response element exhibited marked transcriptional activation when supplemented with pyruvate or each of the three BCKAs. These new findings are in line with earlier Western analyses using human cancer cell lines that report stabilization of endogenous HIF‐1α protein upon treatment with these same compounds (Lu *et al*., 2005). Our studies extend these findings by revealing that HIF‐1α is not only stabilized, but indeed the holo HIF‐1 transcriptional complex is functionally activated. This is important because, unlike worms, mammalian cells employ a second α‐ketoglutarate‐dependent hydroxylase, factor‐inhibiting HIF‐1 (FIH), that functions as an asparagine hydroxylase and acts like EGL‐9/PHD to inhibit HIF‐1α activity under normoxic conditions. FIH works by preventing HIF‐1α from interacting with the p300 transcriptional co‐activator protein (Hewitson *et al*., 2002). Inhibition of FIH by endogenous citric acid cycle metabolites, including pyruvate, has been previously reported (Rose *et al*., 2011). *C. elegans* lacks FIH, and so if the three BCKAs were exclusively targeting FIH in 3T3‐L1 cells, this would provide a simple explanation for why there was no effect of these compounds on HIF‐1::myc stabilization in worms.

In humans, disruptive mutations of BCKADH result in maple syrup urine disease (MSUD). Patients with MSUD are susceptible to accumulation of large amounts of serum branched‐chain α‐amino acids (BCAAs) and branched‐chain α‐ketoacids (BCKAs), particularly when consuming diets high in protein. If left untreated, patients are at risk of developing cerebral edema (Burrage *et al*., 2014). Even when managed properly, patients with MSUD often suffer intellectual and social impairments (Simon *et al*., 2007). The reasons behind this neurotoxicity are thought to be many but, among others, serum leucine and BCKAs interfere with large neutral amino acid transport across the blood–brain barrier that in turn alters neurotransmitter biosynthesis (Oldendorf & Szabo, 1976). These compounds also inhibit other α‐ketoacid dehydrogenases, including PDH and α‐KGDH (Patel *et al*., 1973; Patel, 1974), which in turn lowers mitochondrial ETC flux. Finally, elevation of 4M2OV contributes to glutamate and aspartate depletion in neurons by sinking away nitrogen (Zielke *et al*., 1997). In this light, life extension in worms is not only quite surprising but makes finding the mechanism(s) by which BCKAs extend lifespan clinically relevant.

Finally, in preliminary studies, we have begun to extend our findings to human mitochondrial diseases. Remarkably, we find that human osteosarcoma cybrid cell lines containing mutant mitochondria derived from patients with Leiber's hereditary optic neuropathy (LHON) also generate an exometabolic profile enriched in α‐ketoacids and α‐hydroxyacids, the same ones found in the exometabolome of Mit mutants (Figs S13 and S14). Just as in worms, addition of FCCP to these cells abrogated accumulation of these compounds. It remains to be determined what role, if any, alterations in α‐ketoglutarate‐dependent hydroxylase activity play in the etiology of human mitochondrial diseases, but this will likely be an exciting area of future investigation.

## Methods

### *Caenorhabditis elegans* strains and maintenance

The following *C. elegans* strains were used for this study: N2 Bristol (wild‐type), CB1141 [*cat‐4(e1141)V*], MQ887 [*isp‐1(qm150)IV*], CL2166 [*(gst‐4::gfp)III*]; SLR0047 [*cat‐4(e1141)V; iaIs28(hif‐1p::hif‐1a::myc + unc‐119(+)*], TJ564 [*isp‐1(qm150)IV;(gst‐4::gfp)III*]; ZG31 [*hif‐1(ia4)V*] and ZG580 [*unc‐119(ed3) III*;* iaIs28(hif‐1p::hif‐1a::myc + unc‐119(+)*]. Construction of ZG580 has been described previously (Zhang *et al*., 2009). SLR0047 was constructed by crossing ZG580 with CB1141. The status of the *unc‐119(ed3)III* allele was not tested. All strains were maintained at 20 °C, on lawns of *E. coli* (OP50) spread onto plates of NGM‐agar, using standard worm culture techniques (Wood, 1988).

### Chemicals and reagents

Supplier and catalogue numbers for all purchased reagents follow: *Sigma‐Aldrich*: β‐alanine (146064), dimethyl 2‐oxoglutarate (349631), fumaric acid (F‐8509), α‐ketoglutarate (disodium salt) (K3752), lactic acid (L1875), 2,4‐pyridinedicarboxylic acid (2,4‐PDA) (04473), sodium 2‐oxobutyrate (K0875), sodium 3‐methyl‐2‐oxobutyrate (198994), sodium (±)‐3‐methyl‐2‐oxovalerate (K7125), sodium 4‐methyl‐2‐oxovalerate (K0629), sodium pyruvate (P2256), succinic acid (S3674), sodium azide (S8032), and glutaric acid (G3407). *Fisher Scientific*: carbonyl cyanide 4‐(trifluoromethoxy) phenylhydrazone (FCCP, #50‐810‐552), 2,4‐PDA (AC13186), and cobalt (II) chloride (AC21413). Mouse monoclonal antibodies targeting c‐*myc* (9E10) and tubulin (E7) were obtained from Developmental Studies Hybridoma Bank (Iowa City, IA, USA). HRP‐labeled anti‐mouse (NA9310) and anti‐rabbit (NA9340) secondary antibodies were purchased from GE Life Sciences (Pittsburgh, PA, USA). Antibodies targeting epigenetic modifications of histone H3 were purchased from Active Motif (Carlsbad, CA)—H3K4me3 (39160), H3K9me3 (39162), H3K27me3 (39537), and H3K36me3 (61102).

### Tissue culture

Mouse 3T3‐L1 fibroblasts (cat # CL‐173) were purchased from the ATCC (Manassas, VA, USA). 3T3‐L1 cells were cultured in DMEM (Sigma, D6429) supplemented with 10% (v/v) bovine calf serum BCS (Hyclone, cat # SH30073.03), 100 μg mL^−1^ penicillin, and 100 μg mL^−1^ streptomycin (Gibco, cat # 10378‐016), at 37 °C in a humidified atmosphere of 5% CO_2_ and ambient O_2_. Cells were passaged by dissociation from growth substrate using 0.05% trypsin‐EDTA solution (Hyclone, cat # SH30236.01).

### Nematode lifespan studies

Lifespan studies described in the main text were undertaken using NGM‐agar plates. Animals of the relevant genotype and developmental stage were transferred to fresh plates supplemented with test compound at the appropriate concentration and incubated at 20 °C. Where specified, plates contained 50 μm 5‐fluorodeoxyuridine to sterilize animals. Plates were spread with OP50 bacteria and allowed to air dry for 18 h at ambient temperature (23 °C) before use. Significance testing for lifespan alteration was calculated using a log‐rank test. A significance threshold of *P* < 0.05 was chosen. Additional lifespan study conditions are described in Supporting Information.

### α‐Ketoacid uptake assay

Age‐synchronized *C. elegans* (120,000 N2 or *cat‐4 (e1141)* worms/sample) were grown to adulthood on standard NGM‐agar/OP50 lawns. Animals were washed from agar plates with S‐Basal media (25 mm K_2_HPO_4_, 25 mm KH_2_PO_4_, and 100 mm NaCl, pH 6.8), and then further washed free of eggs and bacteria with 6 × 50 mL S‐Basal. Sucrose flotation was performed to remove bacteria attached to the worm cuticle (Föll *et al*., 1999), followed by 3 × 15 mL washes with S‐Basal. The total volume of worms + S‐Basal was reduced to 1.5 mL, and animals were transferred to a 3.5‐cm glass dish (custom made, Kimax). To the dish, 3 μL each of 100 mm 2‐OB, 3M2OB, 2M2OV, or 4M2OV was added (final concentrations, 200 μm). Animals were briefly allowed to settle by gravity, and a 100 μL aliquot was then drawn off at *t* = 0 h. The dish was transferred to an orbital shaker and rotated at 100 rpm for 18 h at ambient temperature (23 °C). At the end of this period, the volume in the dish (measured by weight, assuming a density of 1 g mL^−1^) was returned to 1.4 mL by the addition of S‐Basal, to account for evaporative losses. Animals were then removed by centrifugation (3 min, 2000 *g*), and the supernatant was collected and filtered using a 0.20‐μm nylon syringe filter (Life Science Products, Cat. No. 6502‐413X), and then stored at −80 °C ready for quantification. Percent removal of each α‐ketoacid from the supernatant fraction was measured by GC‐MS using identical derivatization and analytical techniques that we described in detail previously for worm exometabolome mapping (Butler *et al*., 2012; Mishur *et al*., 2013).

### ^1^H NMR and chemical stability assays in the presence of *E. coli*

Metabolite stability measurements in the presence or absence of OP50 *E. coli* were analyzed as follows: chemicals of interest were made to 10 mm in S‐Basal (pH 6.8, supplemented with 1 × 10^9^ cfu mL^−1^ OP50 (final concentration) and 10 μL of 5 mg mL^−1^ cholesterol in ethanol) to a final volume of 5 mL. Control solutions lacking OP50 were also prepared. Alternatively, bacteria were treated with either kanamycin (50 μg mL^−1^), tetracycline (5 μg mL^−1^), or *prior* to addition of the metabolite of interest either heated for 30 min at 65 °C or exposed to UV light to cross‐link DNA (5 min, UV Stratalinker 2400, 600 mJoules). Samples were then incubated at 20 °C and 1 mL aliquots then removed at *t* = 0, 24, 48, and 72 h. Bacteria were removed either by passing aliquots through a 0.2‐μm cellulose acetate syringe filter (Nalgene, Fisher Cat. No. 190‐2520), or by centrifugation (17,000 *g*, 15 min, 4 °C). Purified samples were then stored at −80 °C prior to analysis by ^1^H NMR. Spectra were acquired on either a Bruker Avance 500 NMR spectrometer or a Bruker Avance 600 spectrometer using a water‐presaturation pulse sequence. Typical acquisition parameters consisted of: NS = 16, TD = 64k, D1 = 2 s. For some samples, water presaturation was not used—for these, analogous acquisition parameters were used, but with a shorter delay time (D1) of 1 s.

### Quantification of HIF‐1::myc induction in *C. elegans*

ZG580 and SLR0047 were grown to gravid adulthood on NGM‐agar/OP50 plates. Worms were washed off plates using S‐Basal, resuspended in 3.5 mL of the same media supplemented with 10 μg mL^−1^ cholesterol and 1 × 10^9^ cfu mL^−1^ OP50, and then placed in 6‐cm plastic petri dishes at a density of 400 worms per dish (replicate dishes per treatment). Chemicals of interest were added to a final concentration of 40 mm (except fumarate which was added to 10 mm). Sodium hydroxide was used to balance the pH of all chemicals to 7, prior to addition. After incubating worms at 20 °C for 24 h, worms were collected in S‐Basal, washed twice using the same media, then pelleted and snap‐frozen at −80 °C. Hypoxia treatment was used as a positive control for HIF‐1::myc induction, and this was performed by incubating the worm dishes in 3% O_2_ for 6 h immediately before collection. Worm pellets were boiled in 5% SDS lysis buffer (62.5 mm Tris–HCl, pH 6.8), the lysate was centrifuged, and the supernatant collected in fresh tubes. Protein quantification was performed using the bicinchoninic acid (BCA) assay (Pierce, cat # 23225). Samples were analyzed for HIF‐1::myc expression using Western analysis (20 μg total protein/sample, 10% SDS‐PAGE). Nitrocellulose membranes were blocked in 5% milk then probed with an anti‐*c‐myc* primary antibody (diluted 1:500 in TBS‐T, 1% BSA, overnight at 4 °C). Binding of HRP‐labeled anti‐mouse secondary was visualized using ECL reagent (GE Life Sciences, cat # RPN2109). Protein bands were quantified by densitometry using ImageJ software (NIH, Bethesda, MD, USA).

### Hypoxia‐inducible factor‐1 reporter dual luciferase assay

We used a commercially available lentivirus derivative to stably transfect 3T3‐L1 fibroblasts with a firefly luciferase reporter under the control of a HIF‐1 response element (HRE) (pGreenFire1‐HIF1, System Biosciences, catalog # TR026VA‐N). Geneticin (G418) resistance (400 μg mL^−1^) served as the basis for integrant selection. Several clonal isolates were identified that responded to CoCl_2_ addition (a known inducer of HIF‐1 expression), by upregulating luciferase activity. We chose one that showed the strongest luciferase reporter induction (clone #2). This clone was further transfected with a second lentivirus construct encoding a constitutively active *Renilla* luciferase reporter (GeneCopoeia, catalog # LP‐RLUC‐LV105‐0200). Puromycin (2 μm) resistance served as the basis of integrant selection. Several clonal isolates were screened for dual reporter activity, and the one with the most robust activation following CoCl_2_ addition was selected for further experiments (referred to hereafter as 3T3‐L1‐HRE‐*LucD*). For luciferase activity measurements, 3T3‐L1‐HRE‐*LucD* cells were plated in 96‐well plates (5000 cells well^−1^) and allowed to grow overnight in complete media (DMEM + 10% FCS + antibiotics). After overnight incubation, the media were replaced with Kreb's buffer (formulation KR3 as described by Lelong *et al*. (Lelong & Rebel, 1998)) supplemented with 5 mm glucose and containing one of various metabolites of interest at the indicated concentration, then incubated for 20 h at 37 °C. At the end of this time, luciferase activity was measured using the ‘Dual Luciferase Reporter Kit’ (catalog # E1910) from Promega (Madison, WI). Luminescence was measured on a PerkinElmer Victor3 reader equipped with two automatic injectors.

### Histone methylation Western analysis

Worms were seeded on NGM‐agar/OP50 plates as arrested L1s and then allowed to mature to the L3, L4, young adult or gravid adult stage before being collected and washed with S‐Basal. Worm samples were pelleted by brief centrifugation, snap‐frozen at −80 °C, and then lysed in boiling 5% SDS for 5 min. Cuticle remnants were removed by centrifugation (17,000 *g*, 10 min, 25 °C) and then protein quantification performed using BCA reagent. Protein samples (8 μg) were separated by SDS‐PAGE (4–12% NuPage Bis‐Tris, Invitrogen, cat. # NP0322), transferred to nitrocellulose membranes (5% milk blocking reagent), and then probed using antibodies targeting various histone H3 modifications (diluted 1:1000 in TBS‐T, 1% BSA, overnight at 4 °C). Data across triplicate experiments was normalized to total H3 amounts present in each developmental stage.

### JMD2A inhibition assay

JMJD2A inhibition studies were undertaken using the ‘Demethylase (Jumonji‐type) Enzyme Assay Kit’ (catalog # 700360) from Cayman Chemical (Ann Arbor, MI). Briefly, various chemicals of interest were dissolved in ‘assay buffer’ (specifications provided by manufacturer) to a 100 mm stock concentration. The final concentration of each chemical in the inhibition assay was 10 mm. The final concentration of α‐ketoglutarate in the JMJD2A reaction mix was 0.5 mm, which is an 80‐fold excess over the K^app^_M_ for this obligate co‐substrate (6 μm (Hopkinson *et al*., 2013)). The activity assay of this kit utilizes formaldehyde generated during the demethylation of a histone H3 trimethyl Lys9 peptide substrate to produce a fluorescent product that can be measured by a plate reader. Fluorescence measurements were recorded using a Spectramax M2 fluorescence plate reader at 370‐nm excitation and 465‐nm emission. To ensure that the inhibitory effect of pyruvate was specific for JMJD2A, and not for the downstream formaldehyde detection reaction, we mixed pyruvate directly with our formaldehyde detection reagents and saw no reduction in fluorescence (Fig. S15).

### JMJD‐2 homology modeling

We used Swiss Model (Guex & Peitsch, 1997; Arnold *et al*., 2006) and PDB entry 2YBP (Chowdhury *et al*., 2011) to homology model JMJD‐2 of *C. elegans* (Wormbase cosmid ID: Y48B6A.11). 2YBP is an X‐ray crystal structure of JMJD2A (αα 7‐355, 2.02 Å), the human ortholog of worm JMJD‐2. In the crystal structure, JMJD2A was complexed with R‐2‐hydroxyglutarate in place of α‐ketoglutarate, Ni(II) in place Fe(II) in the active site, and a histone H3K36me3 substrate peptide (αα 30‐41). The QMEAN *Z*‐score for the final model was −1.98. In the 2YBP structure, S288 is known to be displaced relative to the structure with either succinate or α‐ketoglutarate in the active site (Fig. 2 (Chowdhury *et al*., 2011). We selected Chain A as the modeling template, and although the automatic alignment of residues αα 232–244 of JMJD‐2 could have been optimized further, the core Cupin barrel was well matched and sufficient for our purposes. In the final JMJD‐2 structure shown in Fig. 4A, Chain B of 2YBP was automatically chosen by the Magic Fit algorithm for image overlay. Corresponding active site residues are as follows: (human/worm pairs) Y132/Y213, Y177/Y254, F185/F262, H188/H265, N198/N275, K206/K283, W208/W285, H276/H356, and S288/S368.

### Oxygen consumption

Oxygen consumption by N2 and *isp‐1(qm150)* worms (1st day and 4th day adults, respectively) was measured using a Clarke‐type oxygen electrode (Oxygraph plus, Hansatech Instruments, UK). Briefly, following growth on NGM‐agar, 1–2000 worms were resuspended in 1 mL S‐Basal and added to the electrode chamber. FCCP (5 mm in ethanol) and sodium azide (20 mm) were used at final concentrations of 22.5 and 400 μm, respectively. Duplicate, independent experiments were performed.

### Complex V activity assay

To test for inhibitory effects of 3M2OB, 3M2OV, and 4M2OV on complex V activity in bovine heart mitochondria extracts, we used the MitoTox^™^ Complex V OXPHOS activity Microplate Assay kit from Abcam (ab109907). α‐ketoglutarate and glutarate were included as controls. Ten‐fold serial dilutions (10 mm–100 μm) of each compound were tested in duplicate, following the manufacturer's instructions.

## Funding info

Financial support was provided by the Ellison Medical Foundation (AG‐NS‐051908, SLR), the NIH [AG‐047561 (SLR), T32 AG021890 (EM), an NRSA fellowship to RJM, and GM‐109434 (YB)], and the Glenn Foundation for Medical Research (EM). Funding sources had no role in study design, data collection, and analysis, decision to publish, or preparation of the manuscript.

## Conflict of interest

The authors declare there are no conflicting interests controlling the publication of this manuscript.

## Supporting information


**Fig. S1** Proton NMR spectrum of 2‐oxobutyrate before and after a 72 h incubation in the presence of growth‐limited ‘fresh’ (2‐day‐old) or ‘old’ (37 day‐old) OP50 bacteria. Peaks of unknown identity in the +72 h samples are marked with a question mark.
**Fig. S2** Proton NMR spectrum of 3‐methyl‐2‐oxobutyrate before and after a 72 h incubation in the presence of growth‐limited ‘fresh’ (2‐day‐old) or ‘old’ (37 day‐old) OP50 bacteria.
**Fig. S3** Proton NMR spectrum of 3‐methyl‐2‐oxovalerate before and after a 72 h incubation in the presence of growth‐limited ‘fresh’ (2‐day‐old) or ‘old’ (37 day‐old) OP50 bacteria.
**Fig. S4** Proton NMR spectrum of 4‐methyl‐2‐oxovalerate before and after a 72 h incubation in the presence of growth‐limited ‘fresh’ (2‐day‐old) or ‘old’ (37 day‐old) OP50 bacteria.
**Fig. S5** Proton NMR spectrum of pyruvate before and after a 72 h incubation in the presence of growth‐limited ‘fresh’ (2‐day‐old) or ‘old’ (37 day‐old) OP50 bacteria.
**Fig. S6** Proton NMR spectrum of lactate before and after a 72 h incubation in the presence of growth‐limited ‘fresh’ (2‐day‐old) or ‘old’ (37 day‐old) OP50 bacteria.
**Fig. S7** Proton NMR spectrum of fumarate before and after a 72 h incubation in the presence of growth‐limited ‘fresh’ (2‐day‐old) or ‘old’ (37 day‐old) OP50 bacteria.
**Fig. S8** Proton NMR spectrum of succinate before and after a 72 h incubation in the presence of growth‐limited ‘fresh’ (2‐day‐old) or ‘old’ (37 day‐old) OP50 bacteria.
**Fig. S9** 2,4‐PDA is stable in the presence of either alive or dead OP50 *E. coli*.
**Fig. S10** 2,4‐PDA stabilizes HIF‐1::*myc* expression.
**Fig. S11** Dietary supplementation of pyruvate and 2M4OV from the L1 larval stage does not extend lifespan.
**Fig. S12** Exometabolites delay growth of *C. elegans*.
**Fig. S13** Transmitochondrial osteosarcoma cybrid cell lines homoplasmic for either wild‐type mtDNA, mutant G3460A (ND1) mtDNA, or G11778A (ND4) mtDNA, were cultured in the presence or absence of 1μM FCCP, and their exometabolome profiles analyzed by GC‐MS.
**Fig. S14** Plot of metabolites that differed significantly across all three cybrid cell lines following FCCP addition in Fig. S13
**Fig. S15.** Pyruvate does not significantly block formaldehyde detection by the JMJD2A fluorescent detector assay.

**Table S1** Survival data for all lifespan studies.

**Table S2** Proton NMR data corresponding to Fig. 3A.

**Table S3** Metabolites known to competitively inhibit α‐ketoglutarate dependent hydroxylases. See also Table 7 in (Rose *et al*., 2011).

**Table S4** Proton NMR data corresponding to Fig. S9.

**Data S1** Materials and methods.
