# Supplementary material for: Mitochondrial metabolites extend lifespan
Source: Aging Cell. 2016 Jan 5;15(2):336–48. doi: 10.1111/acel.12439 (PMC4783347; doi:10.1111/acel.12439)
Supplement: Supplementary file 1 — Fig. S1 Proton NMR spectrum of 2‐oxobutyrate before and after a 72 h incubation in the presence of growth‐limited ‘fresh’ (2‐day‐old) or ‘old’ (37 day‐old) OP50 bacteria. Peaks of unknown identity in the +72 h samples are marked with a question mark. Fig. S2 Proton NMR spectrum of 3‐methyl‐2‐oxobutyrate before and after a 72 h incubation in the presence of growth‐limited ‘fresh’ (2‐day‐old) or ‘old’ (37 day‐old) OP50 bacteria. Fig. S3 Proton NMR spectrum of 3‐methyl‐2‐oxovalerate before and after a 72 h incubation in the presence of growth‐limited ‘fresh’ (2‐day‐old) or ‘old’ (37 day‐old) OP50 bacteria. Fig. S4 Proton NMR spectrum of 4‐methyl‐2‐oxovalerate before and after a 72 h incubation in the presence of growth‐limited ‘fresh’ (2‐day‐old) or ‘old’ (37 day‐old) OP50 bacteria. Fig. S5 Proton NMR spectrum of pyruvate before and after a 72 h incubation in the presence of growth‐limited ‘fresh’ (2‐day‐old) or ‘old’ (37 day‐old) OP50 bacteria. Fig. S6 Proton NMR spectrum of lactate before and after a 72 h incubation in the presence of growth‐limited ‘fresh’ (2‐day‐old) or ‘old’ (37 day‐old) OP50 bacteria. Fig. S7 Proton NMR spectrum of fumarate before and after a 72 h incubation in the presence of growth‐limited ‘fresh’ (2‐day‐old) or ‘old’ (37 day‐old) OP50 bacteria. Fig. S8 Proton NMR spectrum of succinate before and after a 72 h incubation in the presence of growth‐limited ‘fresh’ (2‐day‐old) or ‘old’ (37 day‐old) OP50 bacteria. Fig. S9 2,4‐PDA is stable in the presence of either alive or dead OP50 E. coli. Fig. S10 2,4‐PDA stabilizes HIF‐1::myc expression. Fig. S11 Dietary supplementation of pyruvate and 2M4OV from the L1 larval stage does not extend lifespan. Fig. S12 Exometabolites delay growth of C. elegans. Fig. S13 Transmitochondrial osteosarcoma cybrid cell lines homoplasmic for either wild‐type mtDNA, mutant G3460A (ND1) mtDNA, or G11778A (ND4) mtDNA, were cultured in the presence or absence of 1μM FCCP, and their exometabolome profiles analyzed by GC‐MS. [file ACEL-15-336-s001.pptx]

## Slide 1
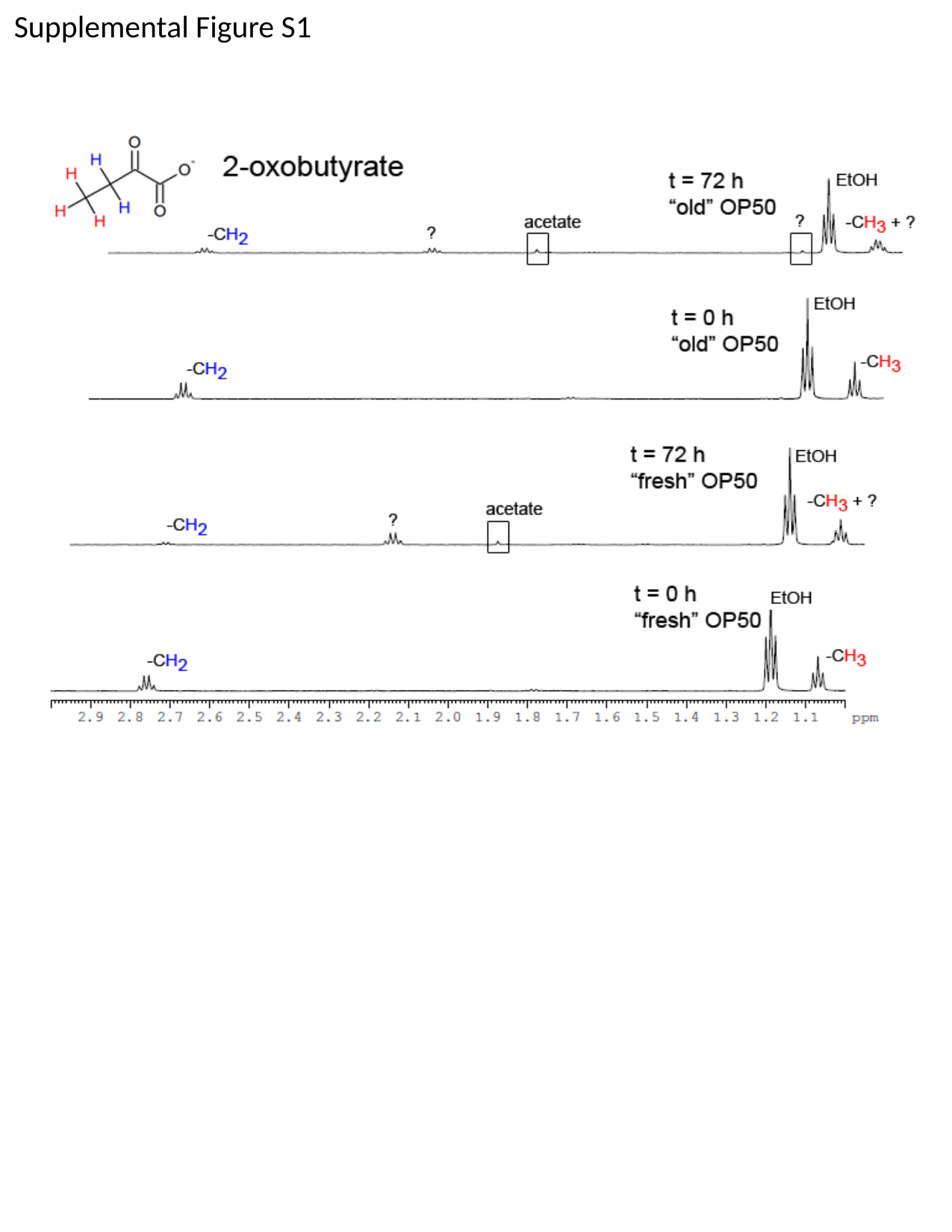

Supplemental Figure S1

## Slide 2
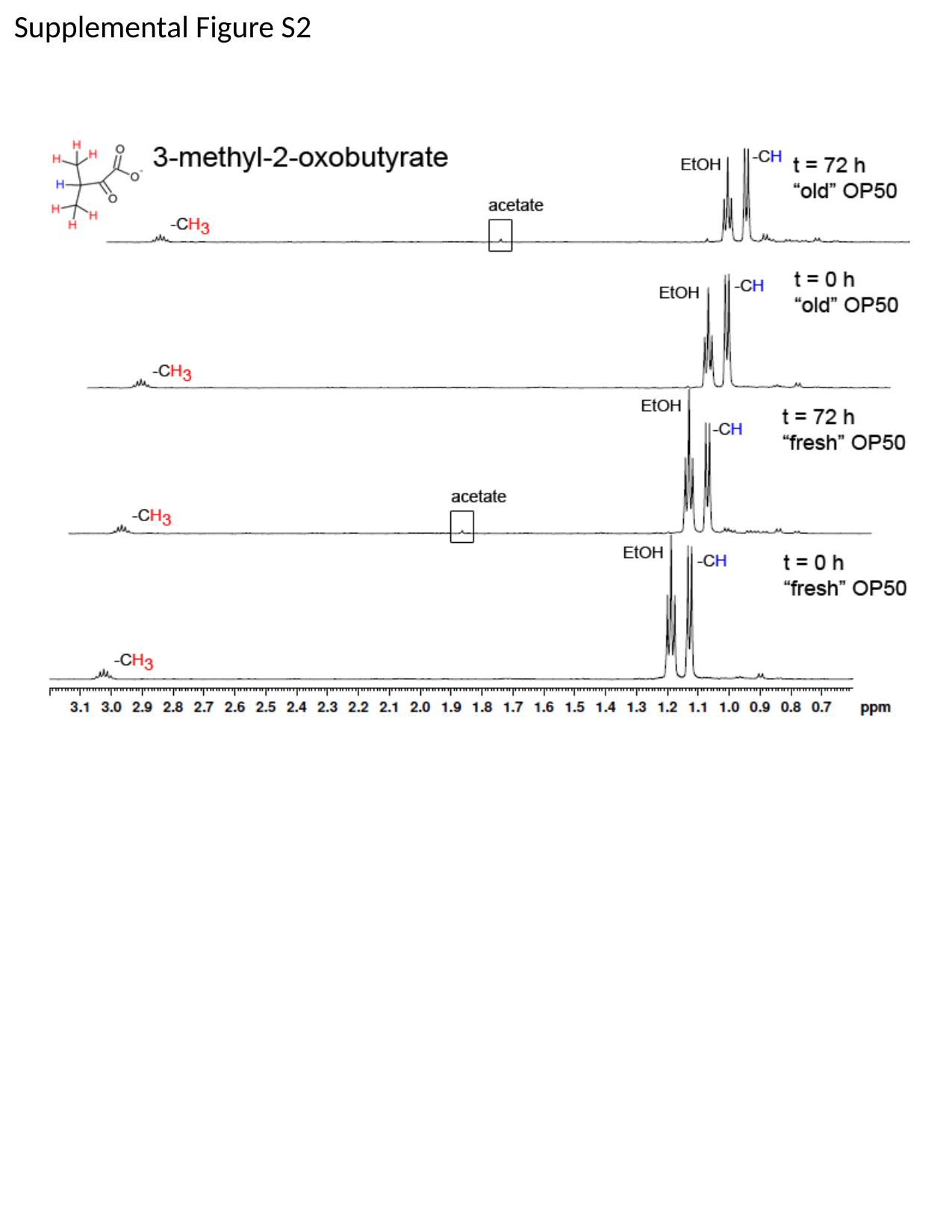

Supplemental Figure S2

## Slide 3
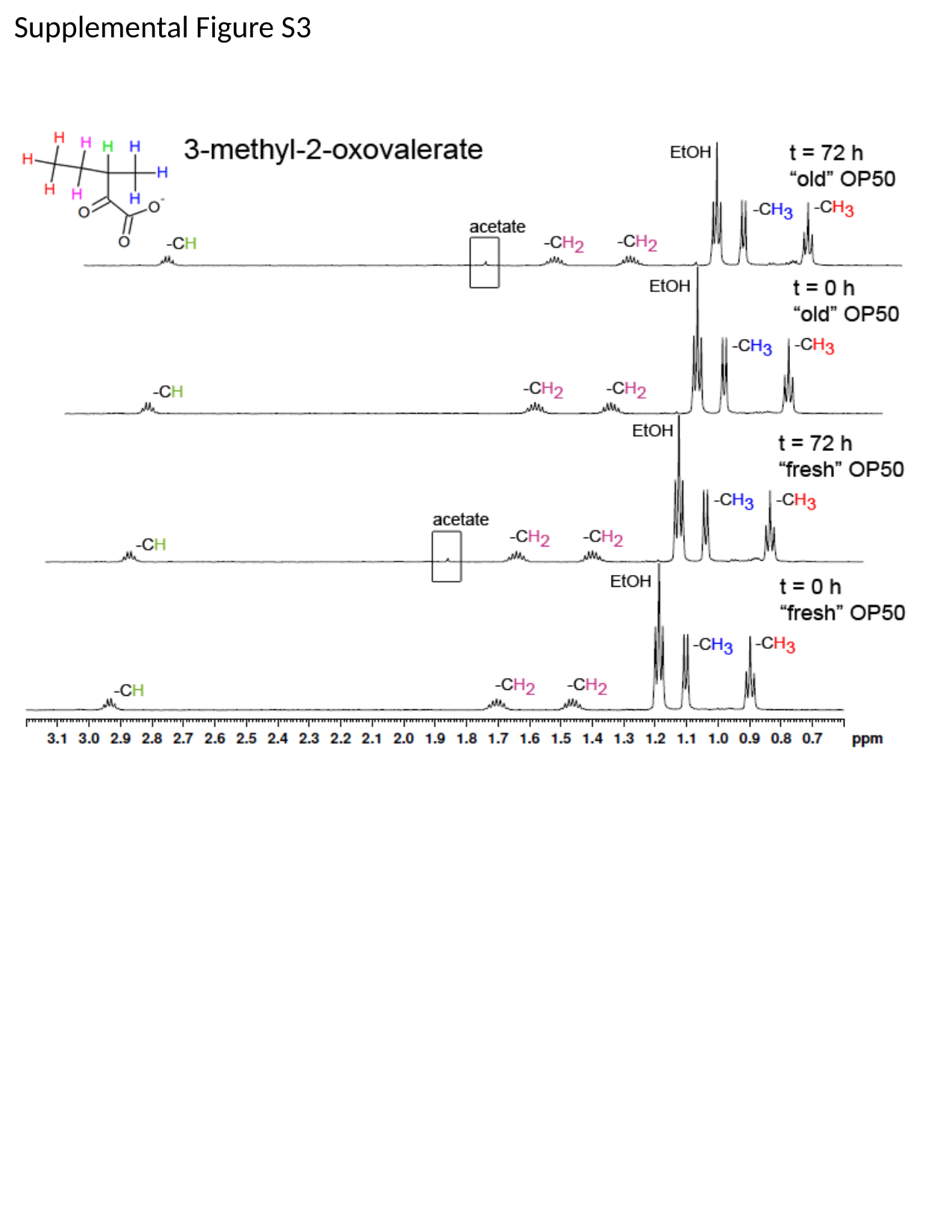

Supplemental Figure S3

## Slide 4
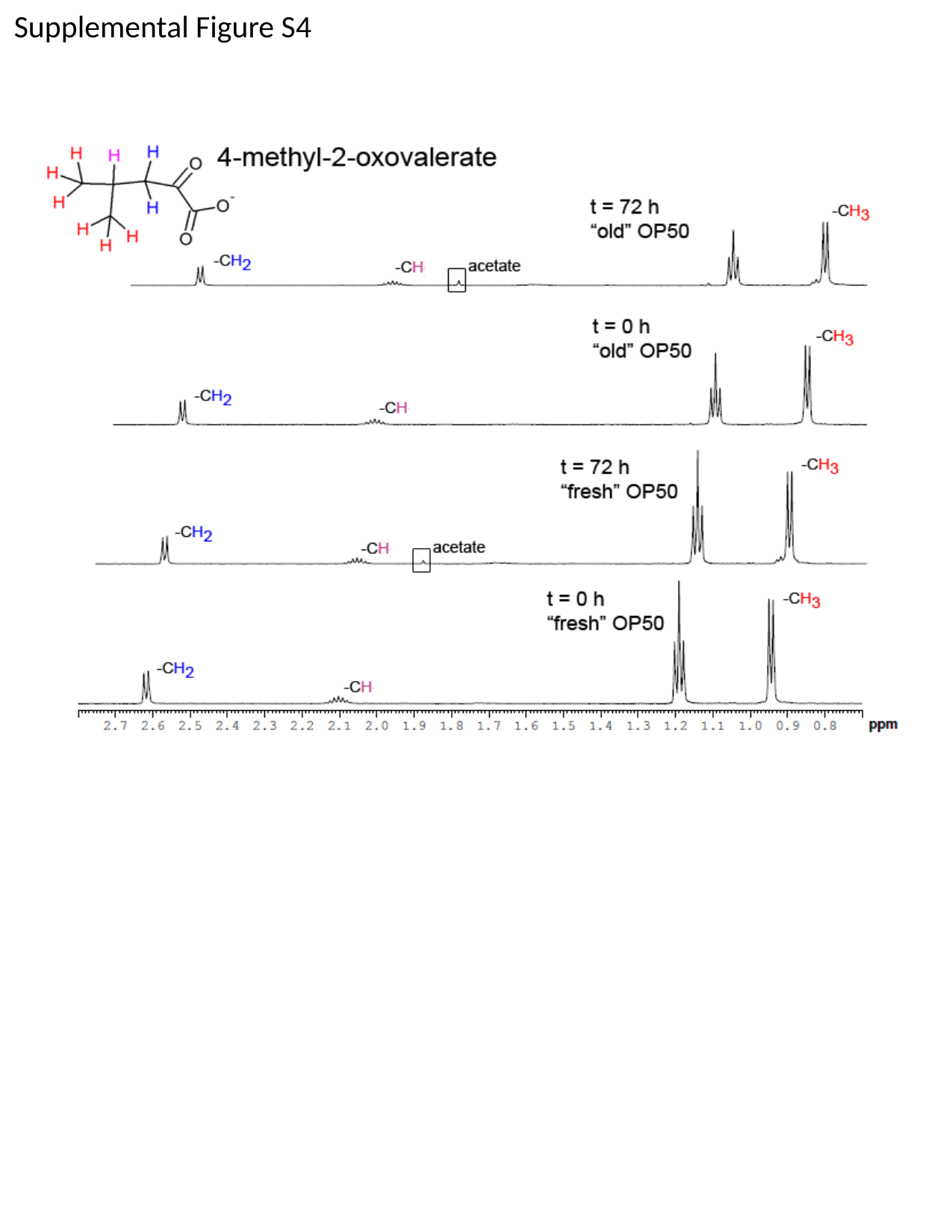

Supplemental Figure S4

## Slide 5
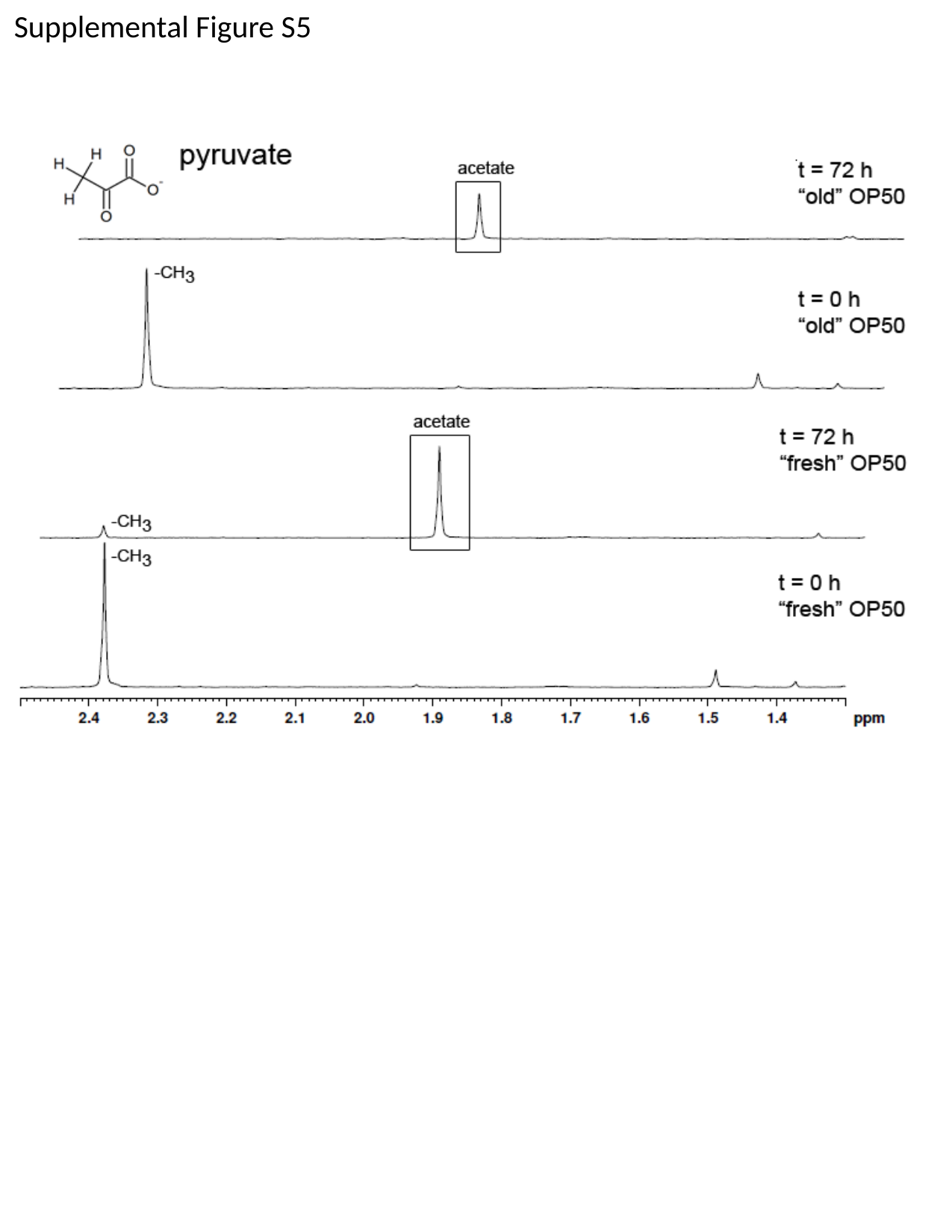

Supplemental Figure S5

## Slide 6
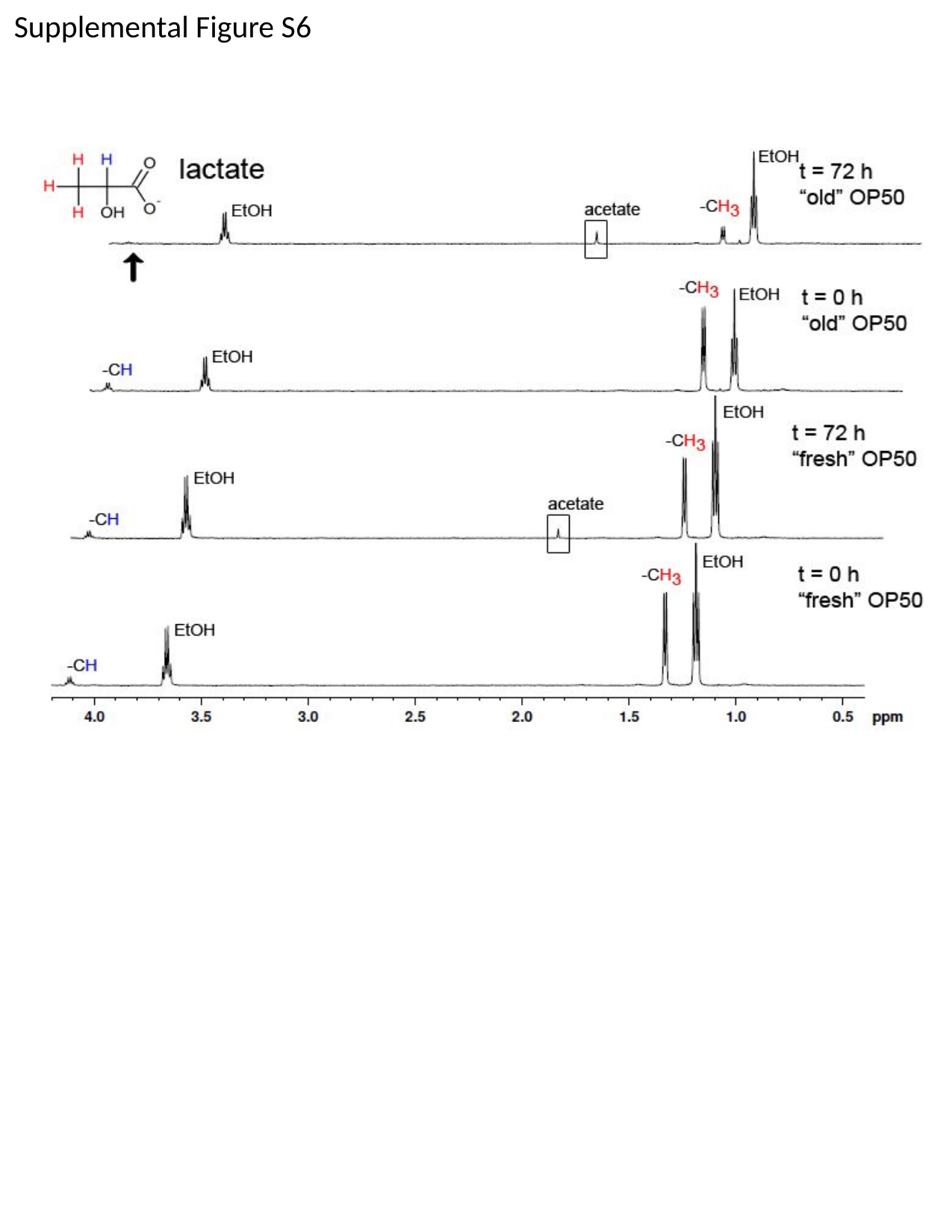

Supplemental Figure S6

## Slide 7
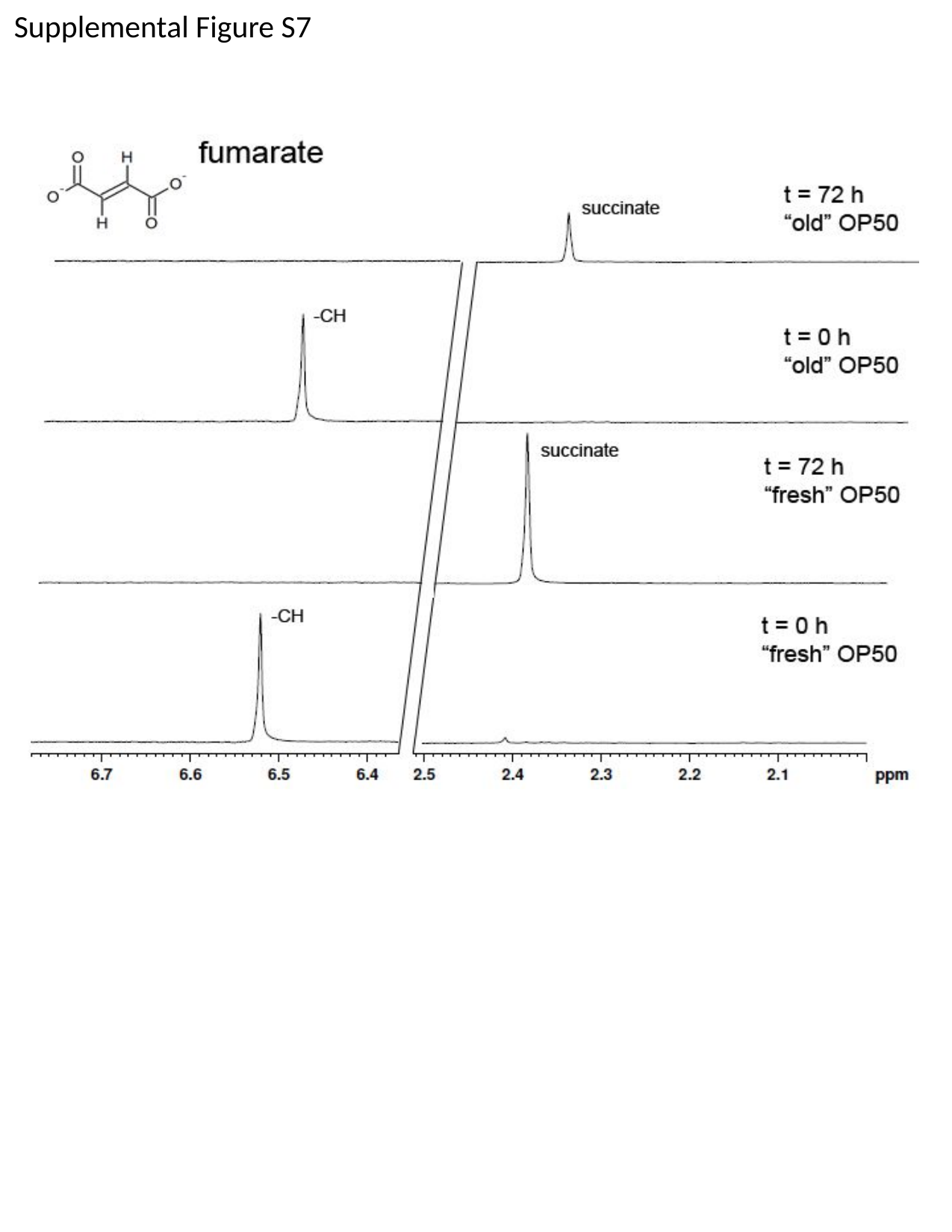

Supplemental Figure S7

## Slide 8
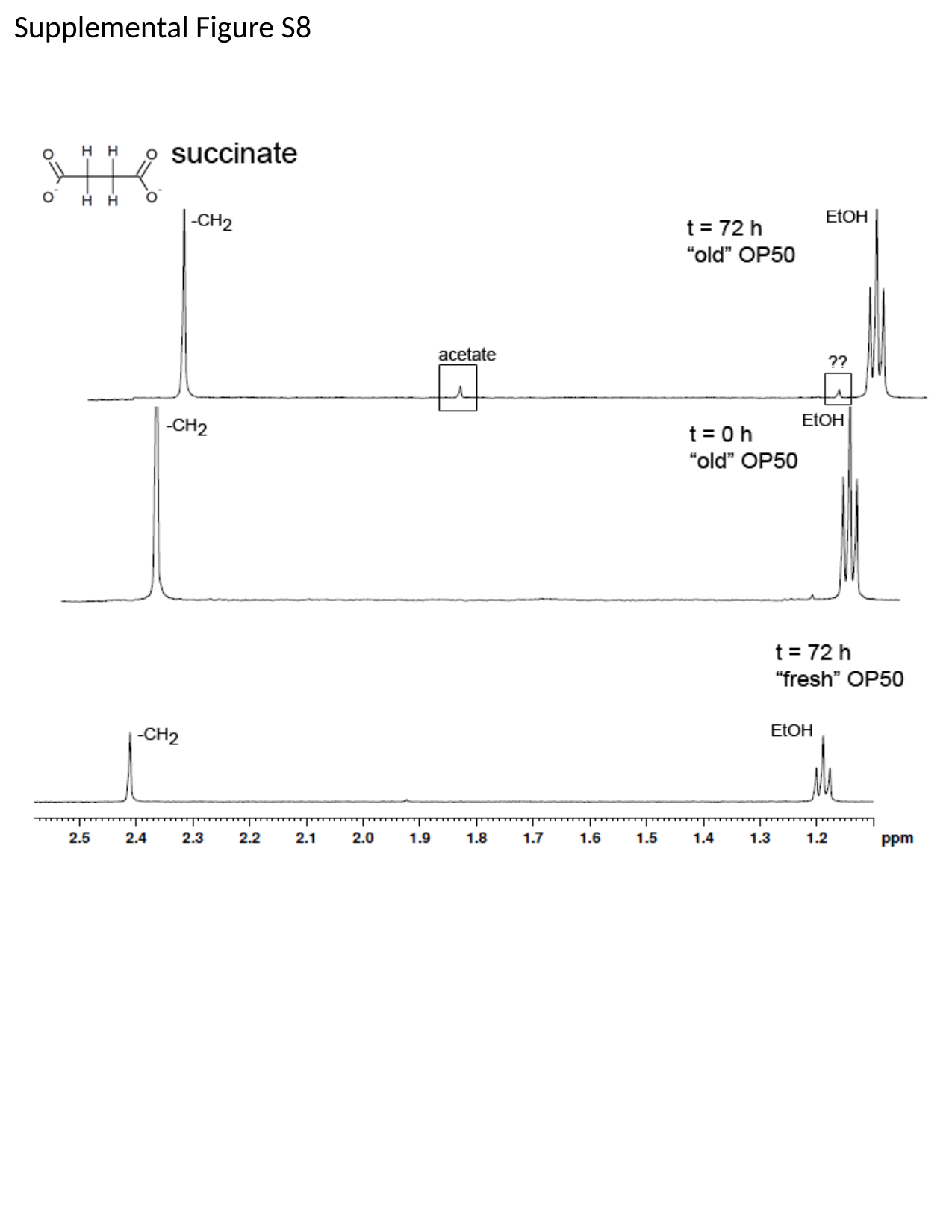

Supplemental Figure S8

## Slide 9
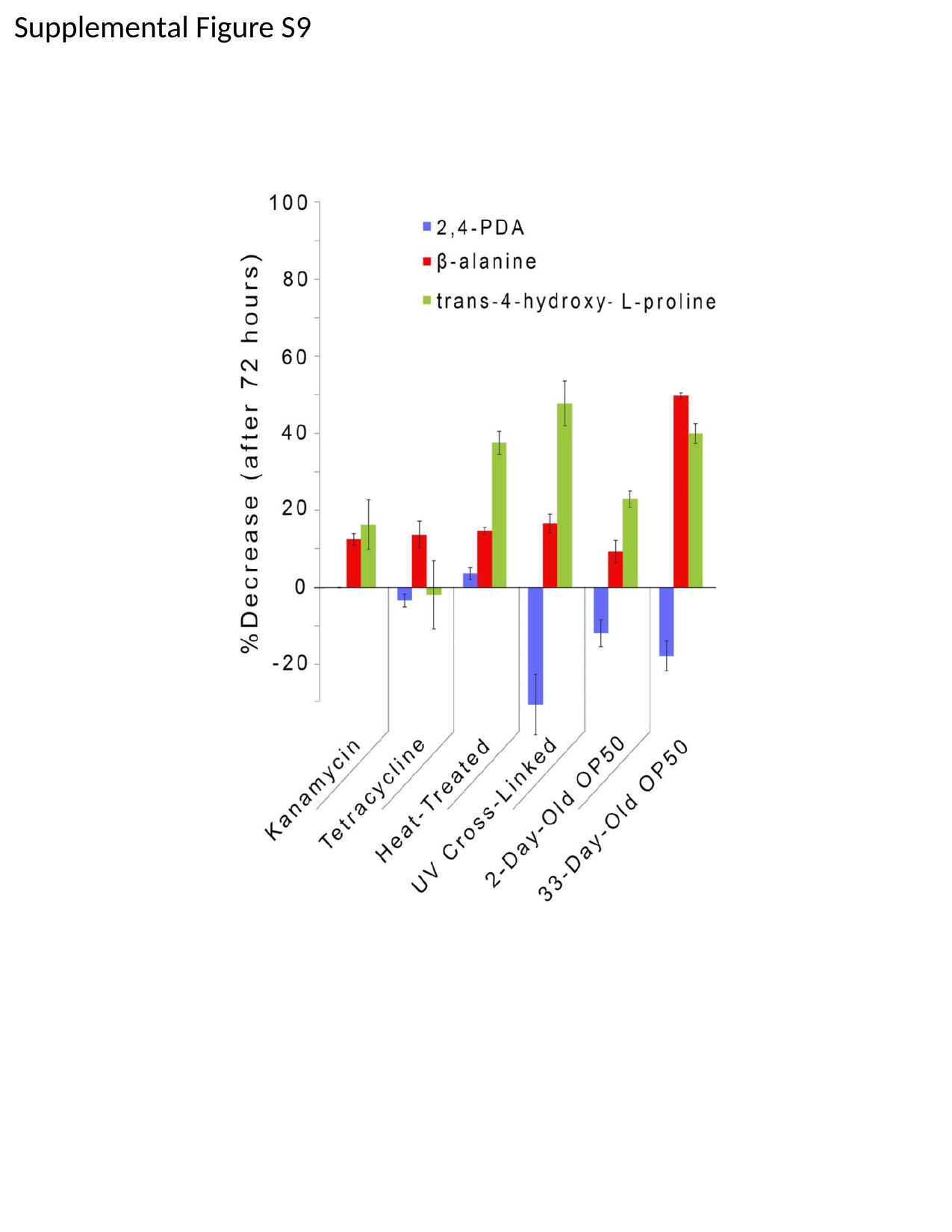

Supplemental Figure S9

## Slide 10
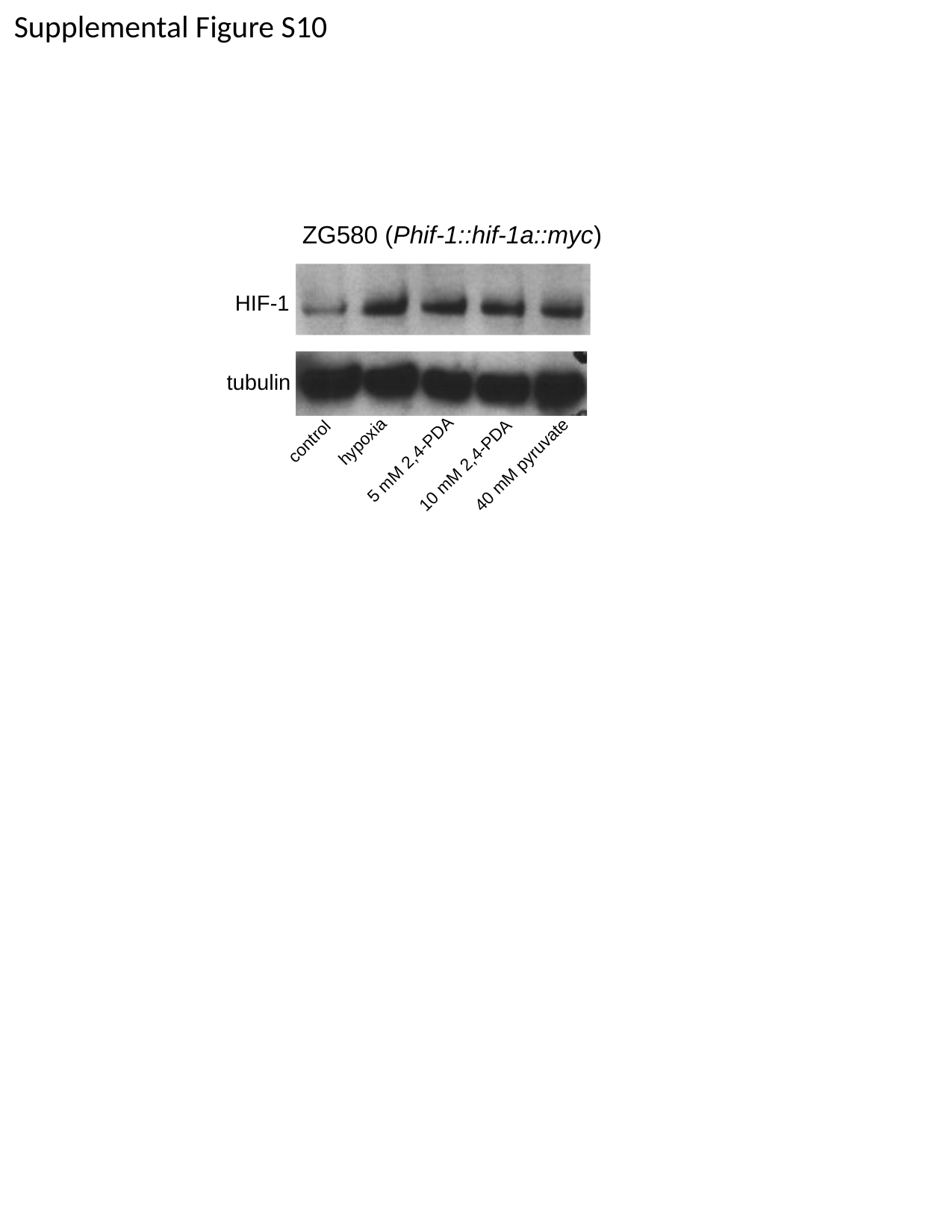

Supplemental Figure S10
ZG580 (Phif-1::hif-1a::myc)
HIF-1
control
hypoxia
tubulin
5 mM 2,4-PDA
10 mM 2,4-PDA
40 mM pyruvate

## Slide 11
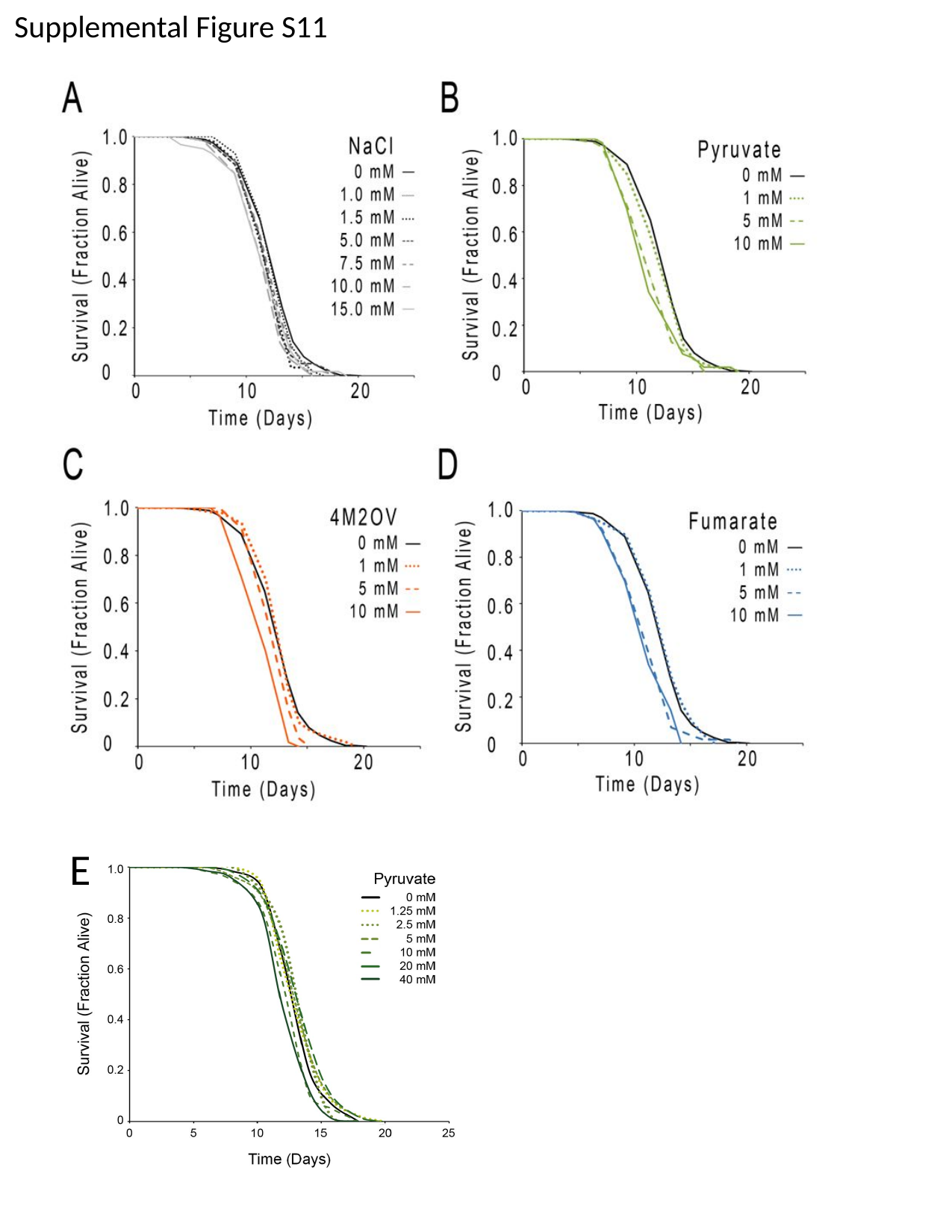

Supplemental Figure S11
E

## Slide 12
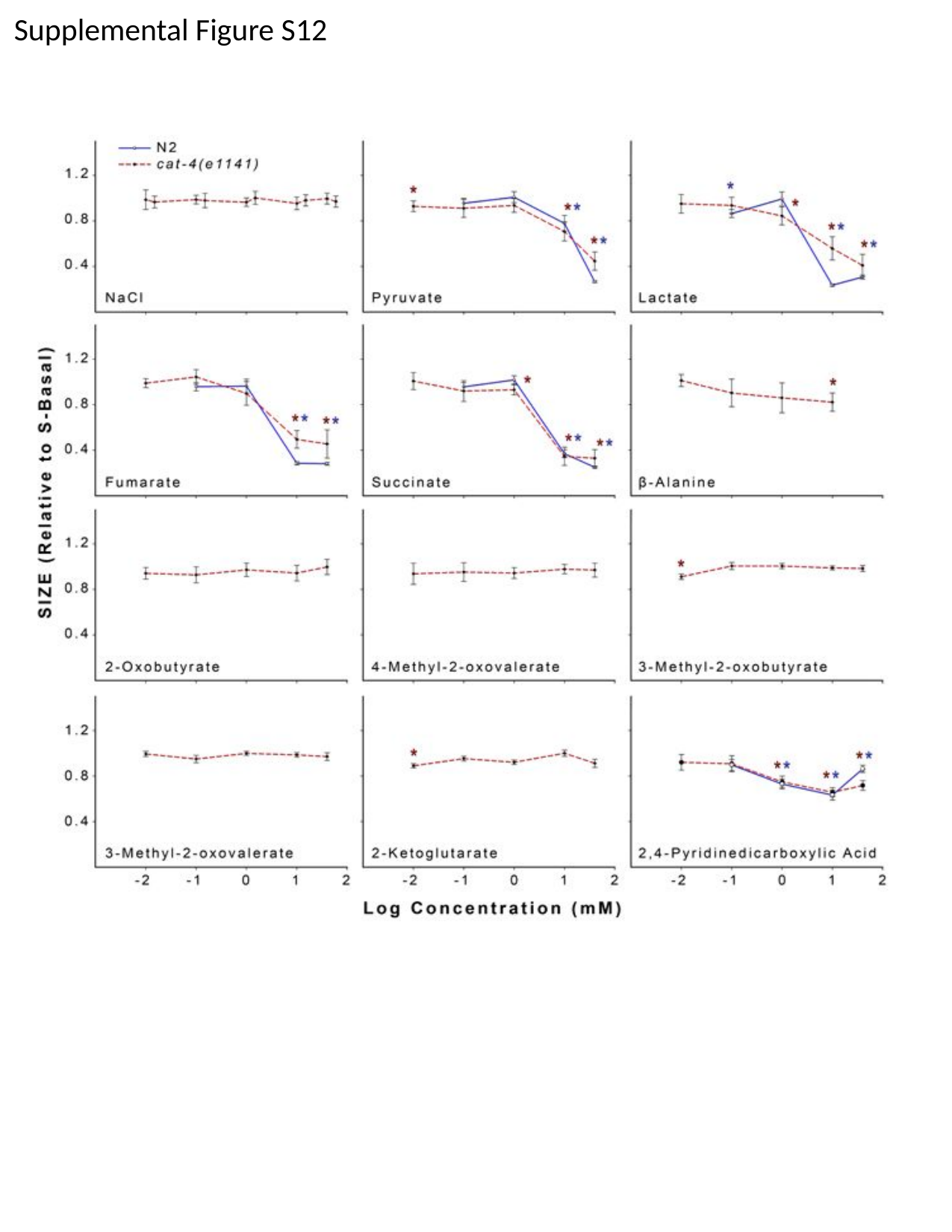

Supplemental Figure S12

## Slide 13
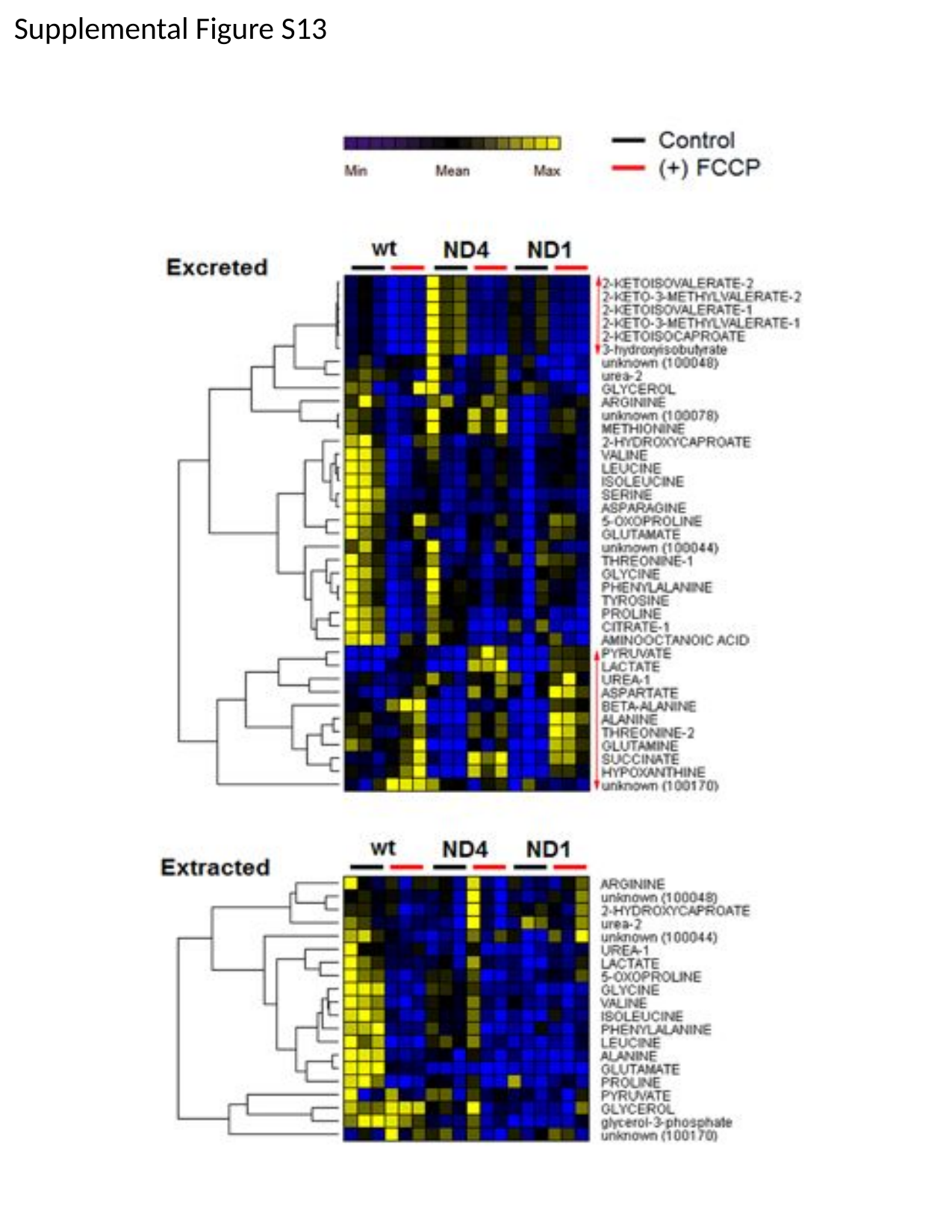

Supplemental Figure S13

## Slide 14
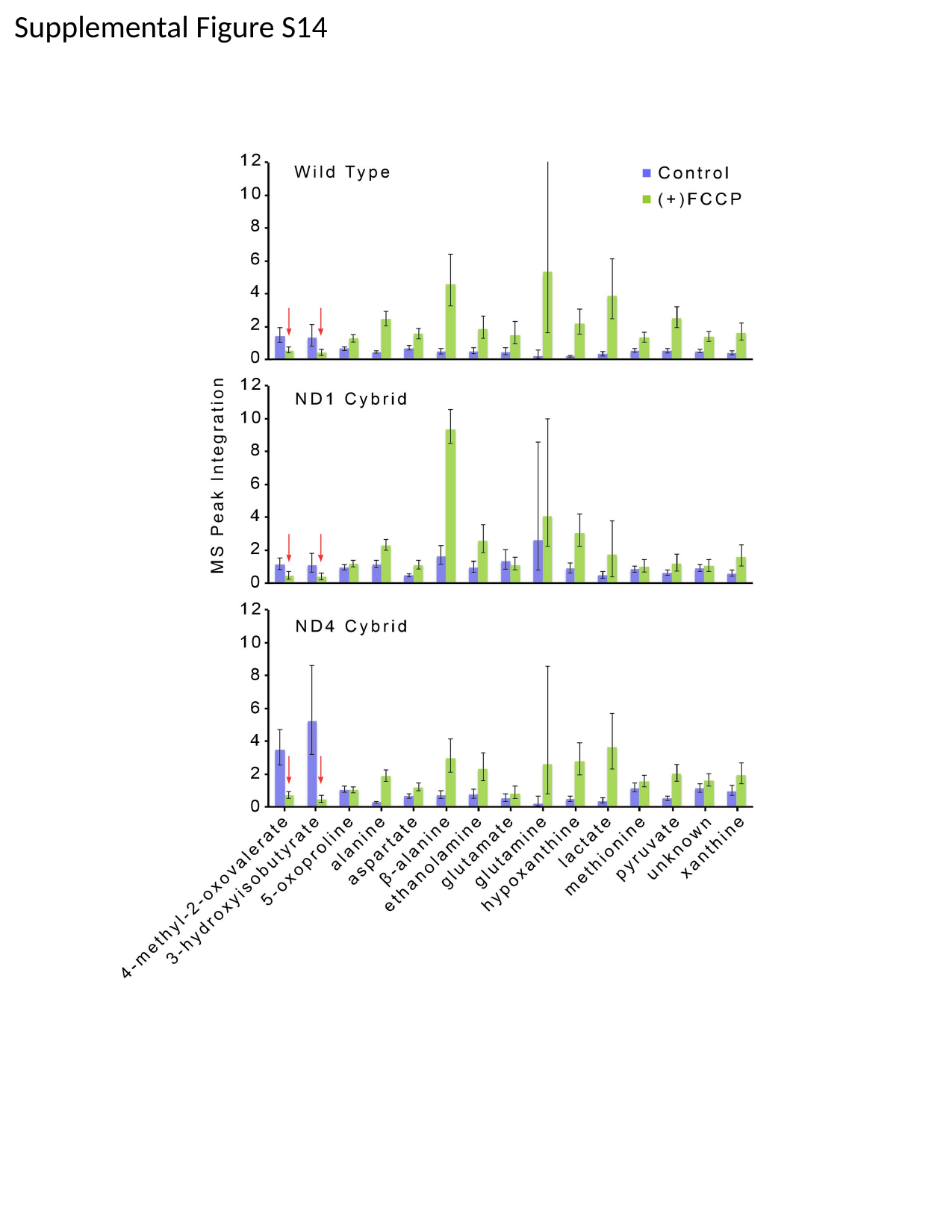

Supplemental Figure S14

## Slide 15
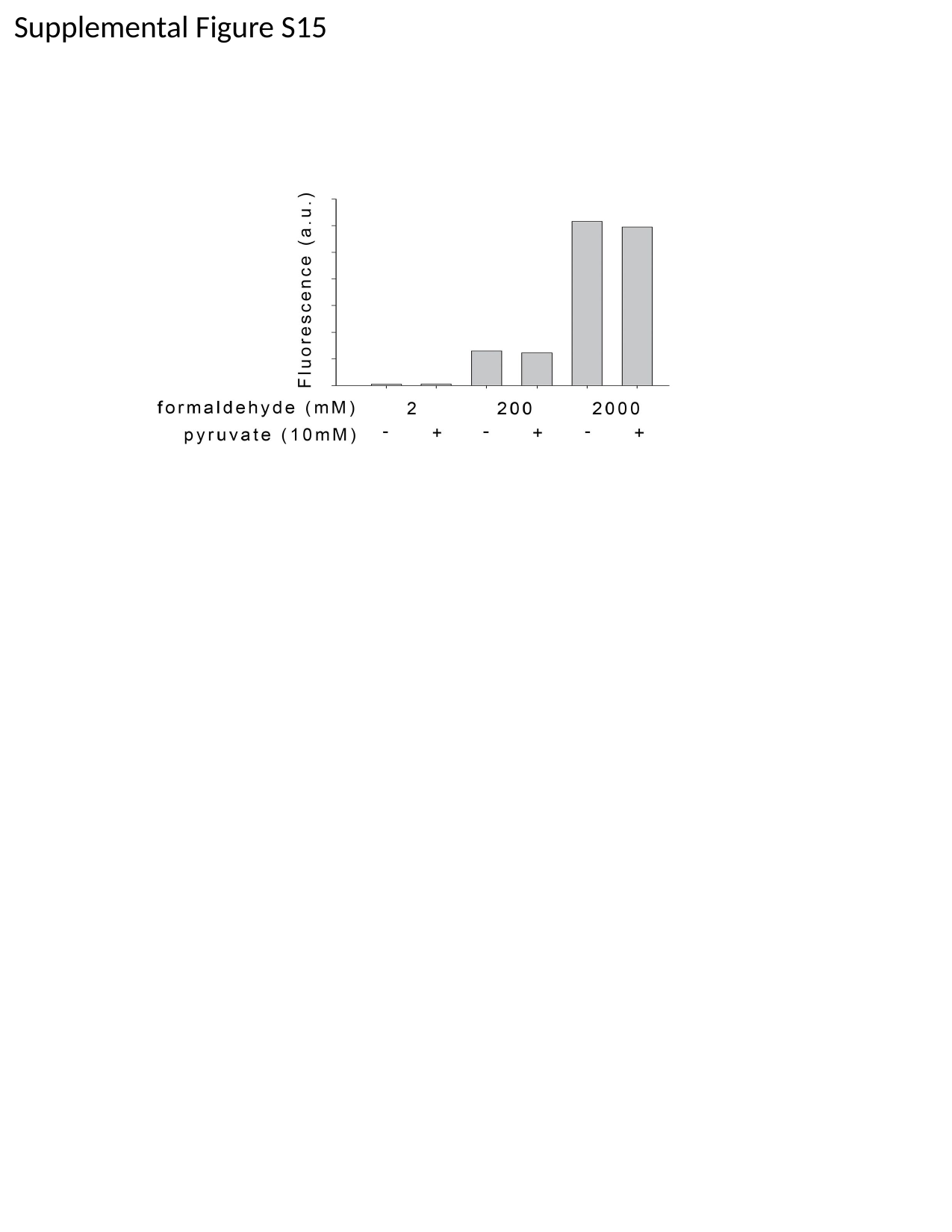

Supplemental Figure S15
