# Supplementary material for: Mitochondrial metabolites extend lifespan
Source: Aging Cell. 2016 Jan 5;15(2):336–48. doi: 10.1111/acel.12439 (PMC4783347; doi:10.1111/acel.12439)
Supplement: Supplementary file 4 — Table S3 Metabolites known to competitively inhibit α‐ketoglutarate dependent hydroxylases. See also Table 7 in (Rose et al., 2011). [file ACEL-15-336-s004.docx]

| **Supplemental Table SIII. Metabolites that are Known to Competitively Inhibit α-ketoglutarate-dependent hydroxylases** | | | | | |
| --- | --- | --- | --- | --- | --- |
| **Compound** | **Cupin Target** | **Species** | **IC_50_ (+/- SEM) (mM)*** | **K_i_ (+/- SEM) (mM)**** | **Reference** |
| **(R)-2HG** | FIH | *Homo sapiens* | 1.5 (0.4) |  | ([*1*](#_ENREF_1)) |
|  | PHD2 | *Homo sapiens* | 7.3 (3.3) |  | ([*1*](#_ENREF_1)) |
|  | JMJD2A | *Homo sapiens* | 0.024 (0.002) |  | ([*1*](#_ENREF_1)) |
|  | JMJD2C | *Homo sapiens* | 0.079 (0.007) |  | ([*1*](#_ENREF_1)) |
|  | FBXL11 | *Homo sapiens* | 0.106 (0.022) |  | ([*1*](#_ENREF_1)) |
|  | ABH2 | *Homo sapiens* | 0.424 (0.077) |  | ([*1*](#_ENREF_1)) |
|  | BBOX1 | *Homo sapiens* | 13.2 (1.1) |  | ([*1*](#_ENREF_1)) |
|  |  |  |  |  |  |
| **(S)-2HG** | FIH | *Homo sapiens* | 0.189 (0.034) |  | ([*1*](#_ENREF_1)) |
|  | PHD2 | *Homo sapiens* | 0.410 (0.15) |  | ([*1*](#_ENREF_1)) |
|  | JMJD2A | *Homo sapiens* | 0.026 (0.003) |  | ([*1*](#_ENREF_1)) |
|  | JMJD2C | *Homo sapiens* | 0.97 (0.024) |  | ([*1*](#_ENREF_1)) |
|  | FBXL11 | *Homo sapiens* | 0.048 (0.015) |  | ([*1*](#_ENREF_1)) |
|  | ABH2 | *Homo sapiens* | 0.15 (0.02) |  | ([*1*](#_ENREF_1)) |
|  | BBOX1 | *Homo sapiens* | 0.142 (0.03) |  | ([*1*](#_ENREF_1)) |
|  |  |  |  |  |  |
| **2-oxobutyrate** | BBOX1 | *Pseudomonas sp. AK1* |  | 1.3 (0.02) | ([*2*](#_ENREF_2)) |
|  | Prolyl-4-hydroxylase | *Gallus gallus* |  | 7.6 | ([*3*](#_ENREF_3)) |
|  |  |  |  |  |  |
| **pyruvate** | BBOX1 | *Pseudomonas sp. AK1* |  | 1.4 (0.3) | ([*2*](#_ENREF_2)) |
|  | Prolyl-4-hydroxylase | *Gallus gallus* |  | 15 | ([*3*](#_ENREF_3)) |
|  |  |  |  |  |  |
|  |  |  |  |  |  |
| **succinate** | BBOX1 | *Pseudomonas sp. AK1* |  | 0.07 (0.01) | ([*2*](#_ENREF_2)) |
|  | Prolyl-4-hydroxylase | Alvinella caudata | 4.5 |  | ([*4*](#_ENREF_4)) |
|  | Prolyl-4-hydroxylase | *Gallus gallus* | 2.5 |  | ([*4*](#_ENREF_4)) |
|  | Prolyl-4-hydroxylase | *Gallus gallus* |  | 0.4 | ([*3*](#_ENREF_3)) |
|  | JMJD2C | Homo sapiens | 0.71 |  | ([*5*](#_ENREF_5)) |
|  |  |  |  |  |  |
| **fumarate** | JMJD2C | Homo sapiens | 1.6 |  | ([*5*](#_ENREF_5)) |
|  |  |  |  |  |  |
| **2,4-pyridine** | BBOX1 | *Pseudomonas sp. AK1* |  | 0.0002 (0.0000) | ([*2*](#_ENREF_2)) |
| **dicarboxylate** | Prolyl-4-hydroxylase | Alvinella caudata | 0.048 |  | ([*4*](#_ENREF_4)) |
|  | Prolyl-4-hydroxylase | *Gallus gallus* | 0.054 |  | ([*4*](#_ENREF_4)) |
|  | JMJD2C | *Homo sapiens* | 0.00066 |  | ([*6*](#_ENREF_6)) |
|  | Prolyl-4-hydroxylase | *Gallus gallus* |  | 0.002 | ([*3*](#_ENREF_3)) |
|  | JMJD2C | Homo sapiens | 0.0014 |  | ([*5*](#_ENREF_5)) |
|  |  |  |  |  |  |

* see relevant reference for specific reaction conditions; ** For relationship between IC_50_ and K_i_ see ([*7*](#_ENREF_7))

1. R. Chowdhury *et al.*, The oncometabolite 2-hydroxyglutarate inhibits histone lysine demethylases. *EMBO reports* **12**, 463 (May 1, 2011).

2. S. F. Ng, H. M. Hanauske-Abel, S. Englard, Cosubstrate binding site of Pseudomonas sp. AK1 gamma-butyrobetaine hydroxylase. Interactions with structural analogs of alpha-ketoglutarate. *The Journal of biological chemistry* **266**, 1526 (Jan 25, 1991).

3. K. Majamaa, H. M. Hanauske-Abel, V. Gunzler, K. I. Kivirikko, The 2-oxoglutarate binding site of prolyl 4-hydroxylase. Identification of distinct subsites and evidence for 2-oxoglutarate decarboxylation in a ligand reaction at the enzyme-bound ferrous ion. *European journal of biochemistry / FEBS* **138**, 239 (Jan 16, 1984).

4. G. Kaule, R. Timpl, F. Gaill, V. Gunzler, Prolyl hydroxylase activity in tissue homogenates of annelids from deep sea hydrothermal vents. *Matrix Biol* **17**, 205 (Jul, 1998).

5. N. R. Rose *et al.*, Inhibitor scaffolds for 2-oxoglutarate-dependent histone lysine demethylases. *Journal of medicinal chemistry* **51**, 7053 (Nov 27, 2008).

6. S. E. Hutchinson *et al.*, Enabling Lead Discovery for Histone Lysine Demethylases by High-Throughput RapidFire Mass Spectrometry. *Journal of biomolecular screening*, (Aug 21, 2011).

7. R. Z. Cer, U. Mudunuri, R. Stephens, F. J. Lebeda, IC50-to-Ki: a web-based tool for converting IC50 to Ki values for inhibitors of enzyme activity and ligand binding. *Nucleic acids research* **37**, W441 (Jul, 2009).
