# Supplementary material for: Mitochondrial metabolites extend lifespan
Source: Aging Cell. 2016 Jan 5;15(2):336–48. doi: 10.1111/acel.12439 (PMC4783347; doi:10.1111/acel.12439)
Supplement: Supplementary file 6 — Data S1 Materials and methods. [file ACEL-15-336-s006.docx]

**SUPPORTING INFORMATION:**

**SUPPLEMENTAL FIGURE LEGENDS:**

**Figure S1.** Proton NMR spectrum of 2-oxobutyrate before and after a 72 hour incubation in the presence of growth-limited ‘fresh’ (2-day-old) or ‘old’ (37 day-old) OP50 bacteria. Peaks of unknown identity in the +72 h samples are marked with a question mark.

**Figure S2.** Proton NMR spectrum of 3-methyl-2-oxobutyrate before and after a 72 hour incubation in the presence of growth-limited ‘fresh’ (2-day-old) or ‘old’ (37 day-old) OP50 bacteria.

**Figure S3.** Proton NMR spectrum of 3-methyl-2-oxovalerate before and after a 72 hour incubation in the presence of growth-limited ‘fresh’ (2-day-old) or ‘old’ (37 day-old) OP50 bacteria.

**Figure S4.** Proton NMR spectrum of 4-methyl-2-oxovalerate before and after a 72 hour incubation in the presence of growth-limited ‘fresh’ (2-day-old) or ‘old’ (37 day-old) OP50 bacteria.

**Figure S5.** Proton NMR spectrum of pyruvate before and after a 72 hour incubation in the presence of growth-limited ‘fresh’ (2-day-old) or ‘old’ (37 day-old) OP50 bacteria.

**Figure S6.** Proton NMR spectrum of lactate before and after a 72 hour incubation in the presence of growth-limited ‘fresh’ (2-day-old) or ‘old’ (37 day-old) OP50 bacteria.

**Figure S7.** Proton NMR spectrum of fumarate before and after a 72 hour incubation in the presence of growth-limited ‘fresh’ (2-day-old) or ‘old’ (37 day-old) OP50 bacteria.

**Figure S8.** Proton NMR spectrum of succinate before and after a 72 hour incubation in the presence of growth-limited ‘fresh’ (2-day-old) or ‘old’ (37 day-old) OP50 bacteria.

**Figure S9. 2,4-PDA is stable in the presence of either alive or dead OP50 *E. coli*.**  2-day old OP50 bacteria were incubated for 72 hrs with 2,4-pyridine dicarboxylic acid (2,4-PDA), β-alanine or trans-4-hydroxy-L-proline. The latter two compounds showed little bacterial metabolism in the presence of fresh bacteria, as measured in **Fig. 3A** of the main text. Bacteria were either pre-treated with heat to lyse cells, or exposed to growth-arresting UV-irradiation, or antibiotics (kanamycin and tetracycline) for the duration of the experiment. Both antibiotics induce bacteriostasis when administered in the short term. 33-day bacteria were included as a positive controls for lysis (see **Fig. 3A** of the main text). Figure is summary of data provided in **Supplemental Table S4**.

**Figure S10. 2,4-PDA stabilizes HIF-1::*myc* expression.** 1 day old adult ZG580 worms were exposed to 5 or 10 mM 2,4-PDA, 40 mM pyruvate for 24 hours, or hypoxia for 6 hours, then HIF-1::*myc* expression quantified by western analysis.

**Figure S11. Dietary supplementation of pyruvate and 2M4OV from the L1 larval stage does not extend lifespan.** *cat-4(e1141)* mutants, which have enhanced cuticular permeability, displayed significant life shortening when incubated with the indicated concentration of test compound. Life statistics are included in Supplemental Table SI.

**Figure S12. Exometabolites delay growth of *C. elegans*.** Wild-type (N2) and *cat-4 (e1141)* worms were exposed to the indicated organic acid, at the marked concentration, from the L1 larval stage. At the end of 72 hours, length measurements were recorded. Mean length data for each condition is normalized against control, buffer-only cultured worms. At least ten animals were measured per condition. (Error bars: +/- S.E.M.). Asterisks indicate significantly different from control worms *(p < 0.05,* Student’s t-test with Holm-Bonferroni correction for multiple testing).

**Figures S13.** Transmitochondrial osteosarcoma cybrid cell lines homoplasmic for either wild-type mtDNA, mutant G3460A (ND1) mtDNA, or G11778A (ND4) mtDNA, were cultured in the presence or absence of 1μM FCCP, and their exometabolome profiles analyzed by GC-MS (*top panel).* The twenty most abundant *intracellular* metabolites detectable using the same method are shown for comparison (*bottom panel).* Data is presented in the form of a hierarchically clustered heat map (Pearson’s correlation coefficient-based), where the relative abundance of each metabolite across independent samples (columns) is colored from lowest (*blue)* to highest (*yellow)*. Top *red* arrow highlights BCKAs; bottom *red* arrow indicates end metabolic state after FCCP addition, indicative of enhanced glycolytic flux ([Si *et al.* 2009](#_ENREF_12)). Refer to **Supplemental Materials & Methods** for growth and GC-MS analysis conditions.

**Figure S14**. Plot of metabolites that differed significantly across all three cybrid cell lines following FCCP addition in **Supplemental Figure S13.** Arrows highlight that direction of change differs from rest of metabolites. A Holm-Bonferroni multiple comparison correction was applied over all metabolites. This was a stringent correction and it should be noted that for many metabolites, including multiple α-ketoacids and α-hydroxyacids, their unadjusted p-values fell below the *p* < 0.05 significance threshold, and as a consequence these metabolites have not been included in this figure.

**Figure S15.** Pyruvate does not significantly block formaldehyde detection by the JMJD2A fluorescent detector assay. Detector reagent was incubated with low to high concentrations of formaldehyde in the presence or absence of 10 mM pyruvate. No significant reduction in fluorescence under any condition was observed.

**SUPPLEMENTAL TABLE LEGENDS:**

**Supplemental Table S1.** Survival data for all lifespan studies.

**Supplemental Table S2.** Proton NMR data corresponding to **Fig. 3A**.

**Supplemental Table S3.** Metabolites known to competitively inhibit α-ketoglutarate dependent hydroxylases. See also Table 7 in ([Rose *et al.* 2011](#_ENREF_10)).

**Supplemental Table S4.** Proton NMR data corresponding to **Supplemental Figure S9.**

.

**SUPPLEMENTAL MATERIALS & METHODS:**

***Nematode Larval Development Assay.*** *C. elegans* can be cultured in a liquid environment using minimal S-Basal media (25 mM K_2_HPO_4_, 25 mM KH_2_PO_4_, and 100 mM NaCl, pH 6.8) supplemented with 10 μg/ml cholesterol and 1×10^9^cfu/ml OP50 *E. coli*. Use of this approach limits the ability of OP50 to multiply and hence was selected to initially test the effects of exogenously added metabolites on the growth of wild-type *C. elegans* from the time of hatching*.* Chemicals of interest were tested as follows: First, a ‘1.1× master mix’ was prepared consisting of 1.1×10^9^cfu/ml OP50 and either N2 or *cat-4 (e1141)* worms (one day arrested L1s) at a concentration of 220 animals/ml. To this mix was added a solution of 5 mg/ml cholesterol in ethanol such that the final concentration of cholesterol was 11 µg/ml. In separate wells of a 96-well plate, 90 µL of the master mix was combined with 10 µL of the chemical(s) of interest (dissolved in S-Basal at 10× final concentration, pH adjusted to 7). Final concentrations in wells were: 20 animals/well, 1× 10^9^cfu/ml OP50, 10 µg/ml cholesterol, and 0.2% v/v ethanol. Animals were incubated at 20°C in a hydrating chamber consisting of a loosely sealed container lined with wet paper towels, then removed at 72 h and photographed. Animal length was quantified using ImageJ and used as a surrogate indicator of larval development. At least ten animals were measured for each tested condition. Data was normalized by dividing average test population worm length by the average length of control animals treated with 10 µL S-Basal alone. Populations that differed significantly were identified using the Student’s t-test (p < 0.05). The Holm-Bonferroni correction was applied to control for multiple testing ([Holm 1979](#_ENREF_4)). ^1^H NMR confirmed that most chemicals were not significantly metabolized by **fresh** OP50 over the course of a three-day experiment (**Supplemental Figures S1-8)**. Fumarate, pyruvate, and 2-oxobutyrate, however, were notable exceptions. Month-old OP50 also resulted in significant consumption of test metabolites under these conditions, likely because many cells had lysed and spilled their enzyme contents (see main text, **Fig. 2C**).

***Additional Lifespan Studies***. For the lifespan studies shown in **Supplemental Figure S11** (see also **Supplemental Table S1** for lifespan statistics), minimal S-Basal media was supplemented with 10 μg/ml cholesterol, the relevant concentration of test metabolite, and 2% agar. OP50 bacteria were spotted densely onto these plates due to the poor media growth conditions, then allowed to air dry for 18 h at ambient temperature (23°C) before use. Worms were transferred to plates at the L1 stage and growth on these plates, even of untreated control animals, was noticeably *shortened*. Significance testing for lifespan alteration was calculated using a Log-rank test. A significance threshold of *p <0.05* was chosen.

***cat-4(e1141)***. The *cat-4 (e1141)* strain was originally identified in a screen for animals with reduced dopamine production and consequently has only 10% of the dopamine level found in wild-type worms ([Sulston *et al.* 1975](#_ENREF_13)). This mutant also exhibits reduced serotonin levels ([Avery & Horvitz 1990](#_ENREF_1)). The *cat-4* locus encodes GTP cyclohydrolase 1, the rate-limiting enzyme in the pathway leading to synthesis of tetrahydrobiopterin (BH_4_). BH_4_ is the pterin cofactor used by tyrosine- and tryptophan hydroxylases which are required for the biosynthesis of dopamine and serotonin, respectively ([Goodwill *et al.* 1997](#_ENREF_3)). BH_4_ is also a cofactor for phenylalanine hydroxylase and nitric oxide synthase. Precisely why *cat-4* *(e1141)* mutants have enhanced cuticle permeability (up to 20 times more than wild-type for multiple biochemicals (SLR, *unpublished observation*) remains obscure but studies on cuticle formation in insects provide a clue: Sclerotization, the process of hardening the new cuticle immediately after a molt, is mediated in insects by the deposition of protein and chitin polymers into the cuticle matrix followed by their subsequent crosslinking with catecholamine-derived quinonoids ([Schaefer *et al.* 1987](#_ENREF_11)). If a similar process occurs in *C. elegans* then it is possible that the low catecholamine levels present in *cat-4 (e1141)* mutants adversely affect their cuticle sclerotization, resulting in enhanced permeability.

***Cybrid Cells*.** Human, transmitochondrial osteosarcoma (143B) cybrid cells lines containing either wild-type mitochondrial DNA (mtDNA) or mutant mtDNA (ND1 or ND4) derived from patients with Leiber’s hereditary optic neuropathy (LHON) have been described ([Park *et al.* 2007](#_ENREF_7)). ND1 and ND4 are homoplasmic for the G3460A and G11778A mtDNA mutations, respectively, that affect their namesake subunits of complex I. Cybrid cells were maintained in DMEM + 10% fetal calf serum (FCS).

***Cybrid Metabolome Quantification***. Intracellular and extracellular metabolite samples were collected from each cybrid cell lines as follows: 3x10^6^ cells containing either wild-type, ND1 or ND4 mtDNA were plated on 10 cm dishes and allowed to grow for 48 hr in DMEM + 10% FCS + antibiotics to 80-90% confluency. At the end of this period, the media was replaced with 4 ml Dulbecco’s Phosphate Buffered Saline (DPBS) or DPBS + 1μM FCCP and then the cells were incubated for a further 6 h at 37°C. After this time, the cell supernatant containing exometabolites was immediately collected, centrifuged to remove loose cells, and then stored at -80°C. To obtain intracellular metabolites, cells were removed from culture dishes by trypsin treatment and suspended in 4 ml DPBS. Cells were immediately transferred to 4 °C, counted, and viability measurements calculated using a Vi-cell Cell Viability Analyzer (Beckman Coulter). Metabolites from the cell pellet were extracted into 100% cold methanol (1 ml) for 24 h, at -80°C. Cell bodies were removed by centrifugation, and the supernatant stored at -80°C. Metabolites were derivatized and analyzed using GC-MS, as previously described ([Mishur *et al.* 2013](#_ENREF_6)).

To identify metabolites that changed significantly with FCCP treatment, raw MS exometabolite levels were natural log transformed (to correct for rightward skewing of values), then normalized by fitting to a regression model with the following fixed effects:

ln(M) = β_0_ + β_1_ ln(IS1) + β_2_ ln(IS2) + β_3_ ln(IS3) + β_4_ ln(CELLCOUNT) + β_5_ random(batch)

### where M represents the raw detected level of the exometabolite of interest in the sample of interest; IS1, IS2, and IS3 are the raw detected levels of the internal standards 3,4-dimethoxybenzoic acid, L-norvaline and 5 mM phenylpyruvic acid in the same sample, CELLCOUNT was the number of cells used to generate the sample containing the metabolite of interest (and was measured using the Vi-cell counter), and the βterms represent the final weightings of the factors. The random batch effect is a categorical variable representing the date on which the measurement was taken. The residuals for each exometabolite were used as the normalized response variable in all subsequent analyses. The following regression model was then fit for each exometabolite, with the purpose of identifying exometabolites which had a significant response to FCCP and whether the response of each metabolite was affected by the ND1 or ND4 mutations while taking into account batch effects (day of assay):

### Y = β_0_ + β_f_ FCCP + β_gND1_ ND1 + β_gND2_ ND4 + β_1,gND1_ ND1:FCCP + β_1,gND2_ ND4:FCCP + random(batch)

### The symbols in the above model have the following interpretation:

| Symbol | Interpretation |
| --- | --- |
| Y | Normalized level of the given metabolite |
| FCCP | Dummy variable indicating presence of FCCP in the sample |
| ND1 | Dummy variable indicating whether the cells in the sample contained the ND1 mutation |
| ND4 | Dummy variable indicating whether the cells in the sample contained the ND4 mutation |
| ND1:FCCP | Dummy variable indicating joint presence of FCCP and the ND1 mutation |
| ND4:FCCP | Dummy variable indicating joint presence of FCCP and the ND4 mutation |
| β_0_ | Intercept, i.e. the baseline level of exometabolite Y in the control group |
| β_f_ | Coefficient representing the estimated effect of FCCP on levels of exometabolite Y in WT cells |
| β_gx_ | Coefficient representing the estimated effect of genotype X on levels of exometabolite Y in untreated cells |
| β_f,gx_ | Coefficient representing the estimated difference in the FCCP response between WT cells and cells with genotype X. |

For each metabolite, the estimated coefficients (except for the intercept) were divided by their standard errors and a Wald test was performed (with degrees of freedom estimated from the mixed-effect model) on the resulting test statistic to test the null hypothesis of no difference. A Holm-Bonferroni multiple comparison correction was applied over all metabolites, for all coefficients ([Holm 1979](#_ENREF_4)). **This was a stringent correction and it should be noted that many unadjusted p-values fell below the *p* < 0.05 significance threshold, including multiple α-ketoacids and α-hydroxyacids.** Back-transforming the β coefficients to a linear scale produced estimates of the *fold* change associated with their respective variable (relative to baseline). This data was used to generate **Supplemental Figure S13**. The nlme package of R was used for the above analyses ([Pinheiro *et al.* 2012](#_ENREF_8)). The theory underlying linear mixed-effect models has been described in detail previously ([Davidian & Giltinan 1998](#_ENREF_2); [Lindstrom & Bates 1998](#_ENREF_5); [Pinheiro 2000](#_ENREF_9)).

**SUPPLEMENTAL REFERENCES:**

Avery L, Horvitz HR (1990). Effects of starvation and neuroactive drugs on feeding in Caenorhabditis elegans. *J Exp Zool*. **253**, 263-270.

Davidian M, Giltinan DM (1998). *Nonlinear models for repeated measurement data.* Boca Raton, Florida: CRC Press (First published by Chapman & Hill).

Goodwill KE, Sabatier C, Marks C, Raag R, Fitzpatrick PF, Stevens RC (1997). Crystal structure of tyrosine hydroxylase at 2.3 A and its implications for inherited neurodegenerative diseases. *Nat Struct Biol*. **4**, 578-585.

Holm S (1979). A simple sequentially rejective multiple test procedure. *Scand J Stat* **6**, 65-70.

Lindstrom MJ, Bates DM (1998). Newton-Raphson and EM Algorithms for Linear Mixed-Effects Models for Repeated-Measures Data. *Journal of the American Statistical Association*.

**Vol. 83, No. 404, Dec., 1988**, 1014-1022.

Mishur RJ, Butler JA, Rea SL (2013). Exometabolomic Mapping of Caenorhabditis elegans – A Tool to Non-Invasively Investigate Aging. In *Methods in Molecular Biology*. (T Tollefsbol, ed): Humana Press, Springer Science.

Park JS, Li YF, Bai Y (2007). Yeast NDI1 improves oxidative phosphorylation capacity and increases protection against oxidative stress and cell death in cells carrying a Leber's hereditary optic neuropathy mutation. *Biochimica et Biophysica Acta*. **1772**, 533-542.

Pinheiro J, Bates D, DebRoy S, Sarkar D (2012). nlme: Linear and Nonlinear Mixed Effects Models.ed^eds).

Pinheiro JC (2000). *Mixed- Effects Models in S and S- Plus* New York: Springer, US.

Rose NR, McDonough MA, King ON, Kawamura A, Schofield CJ (2011). Inhibition of 2-oxoglutarate dependent oxygenases. *Chem Soc Rev*. **40**, 4364-4397.

Schaefer J, Kramer KJ, Garbow JR, Jacob GS, Stejskal EO, Hopkins TL, Speirs RD (1987). Aromatic cross-links in insect cuticle: detection by solid-state 13C and 15N NMR. *Science*. **235**, 1200-1204.

Si Y, Shi H, Lee K (2009). Metabolic flux analysis of mitochondrial uncoupling in 3T3-L1 adipocytes. *PLoS One*. **4**, e7000.

Sulston J, Dew M, Brenner S (1975). Dopaminergic neurons in the nematode Caenorhabditis elegans. *J Comp Neurol*. **163**, 215-226.
